# Ligiamycins A and B, Decalin-Amino-Maleimides from the Co-Culture of *Streptomyces* sp. and *Achromobacter* sp. Isolated from the Marine Wharf Roach, *Ligia exotica*

**DOI:** 10.3390/md20020083

**Published:** 2022-01-18

**Authors:** Hyung-Ju Lim, Joon Soo An, Eun Seo Bae, Eunji Cho, Sunghoon Hwang, Sang-Jip Nam, Ki-Bong Oh, Sang Kook Lee, Dong-Chan Oh

**Affiliations:** 1 Natural Products Research Institute, College of Pharmacy, Seoul National University, Seoul 08826, Korea; limju012@snu.ac.kr (H.-J.L.); ahnjunsoo@snu.ac.kr (J.S.A.); ddol1289@snu.ac.kr (E.S.B.); sunghooi@snu.ac.kr (S.H.); sklee61@snu.ac.kr (S.K.L.); 2 Department of Agriculture Biotechnology, College of Agriculture and Life Sciences, Seoul National University, Seoul 08826, Korea; eunji525@snu.ac.kr (E.C.); ohkibong@snu.ac.kr (K.-B.O.); 3 Department of Chemistry and Nanoscience, Ewha Womans University, Seoul 03760, Korea; sjnam@ewha.ac.kr

**Keywords:** co-culture, *Streptomyces*, *Achromobacter*, structure elucidation, *Ligia exotica*, optical rotation, cytotoxicity, antibacterial, decalin, maleimide

## Abstract

*Streptomyces* sp. GET02.ST and *Achromobacter* sp. GET02.AC were isolated together from the gut of the wharf roach, *Ligia* *exotica*, inhabiting the intertidal zone of the west coast of Korea. The co-cultivation of these two strains significantly induced the production of two new metabolites, ligiamycins A (**1**) and B (**2**), which were barely detected in the single culture of *Streptomyces* sp. GET02.ST. The planar structures of ligiamycins A (**1**) and B (**2**) were elucidated as new decalins coupled with amino-maleimides by the analysis of various spectroscopic data, including nuclear magnetic resonance (NMR), ultraviolet (UV), and mass (MS) data. The assignment of two nitrogen atoms in amino-maleimide in **1** was accomplished based on ^1^H-^15^N heteroatom single quantum coherence spectroscopy (HSQC) NMR experiments. The relative configurations of the ligiamycins were determined using rotating frame Overhauser effect spectroscopy (ROESY) NMR data, and their absolute configurations were deduced by comparing their experimental and calculated optical rotations. Ligiamycin A (**1**) displayed antibacterial effects against *Staphylococcus aureus* and *Salmonella enterica*, while ligiamycin B (**2**) exhibited mild cell cytotoxicity against human colorectal cancer cells.

## 1. Introduction

Marine microorganisms physically and chemically interact with neighboring microbes. These interactions are regarded as a driving force to produce bioactive secondary metabolites with pharmaceutical potential [1]. Even though mimicking such complex microbial interactions is challenging, co-culturing two microbial strains in a single culture vessel has been continuously utilized to induce the production of previously unreported bioactive natural products, which has contributed to increasing the chemical diversity of marine microbial compounds [2].

The co-cultivation of the marine fungus *Pestalotia* sp. with the bacterial strain *Thalassospira* sp. CNJ328 produced a new chlorinated benzophenone antibiotic, pestalone, as the first natural product by co-culture [3]. The strain *Thalassospira* sp. CNJ328 also triggered the production of new cytotoxic diterpenoids, libertellenones A–D from the marine fungus *Libertella* sp. [4]. The production of cyclic lipopeptides, emericellamides A and B, was enhanced by 100 times in the co-culture of the fungus *Emericella* sp. from a marine green alga and the marine bacterium *Salinispora arenicola* [5]. However, these early co-culture experiments among marine microbes did not consider ecological relevance much and randomly mixed two different strains. Relatively recently, it has been realized that co-culturing symbiotic or ecologically relevant microbes more efficiently induces the production of new metabolites that are not produced in single cultures. Microscale co-culture experiments with symbiotic Micromonosporaceae strains from marine sponges and ascidians have revealed that 18.5% of 65 co-cultured strains produce unique metabolites, demonstrating the efficiency of this approach [6]. Subsequent chemical studies have discovered a new multi-glycosylated antibiotic, keyicin, from a co-culture of symbiotic *Micromonospora* and *Rhodococcus* associated with marine invertebrates [7]. A mixed culture of marine bacterial strains isolated together from the same marine sediment sample yielded a cyclic depsipeptide, dentigerumycin E, with a polyketide and nonribosomal peptide hybrid origin [8]. The induction of antibacterial metabolites has been observed by co-culturing *Micromonospora* sp. and *Actinokinespora* sp. associated with sea sponges [9]. The co-cultivation of mangrove endophytically symbiotic fungus *Trichoderma* sp. with marine pathogenic bacterium *Acinetobacter johnsonii* has led to the discovery of new sesquiterpenoids, microsphaeropsisins B and C [10].

Among marine invertebrates, which have been considered to harbor microbes potentially producing bioactive metabolites [11], wharf roaches belonging to order Isopoda in the subphylum Crustacea, have not been chemically explored in much detail. In particular, *Ligia* spp. are globally distributed wharf roaches living on rocks on the seashore [12]. As *Ligia* spp. feed on phytoplanktons and degraded plants in intertidal zones, of which the environments vary extremely by tides and host diverse beneficial and pathogenic microorganisms, they could also be associated with microorganisms potentially biosynthesizing biologically and structurally unique compounds. Previous chemical studies reported that new aspochalasins and cyclopeptide metabolites were produced by *Ligia*-associated *Aspergillus* sp. Z4 as a rare chemical study example for *Ligia* spp., indicating the chemical potential of wharf roach-associated microbes [13,14,15].

In this work, we co-cultivated and chemically analyzed microorganisms isolated from the gut of *Ligia exotica*, collected in an intertidal mudflat on the west coast of the Korean peninsula, and found that the co-culture of *Streptomyces* sp. GET02.ST and *Achromobacter* sp. GET02.AC produced distinct chemical profiles compared to the individual cultures of these strains. Further large culture and chromatographic separation of the distinct metabolites enabled us to elucidate their structures and to evaluate their biological activity. Herein, we report the isolation, structure determination, and bioactivities of these compounds, ligiamycins A (**1**) and B (**2**), from the co-culture of the gut strains of *Ligia exotica*.

## 2. Results and Discussion

### 2.1. Co-Culture of Streptomyces sp. and Achromobacter sp. from Ligia exotica

**Figure 1:**
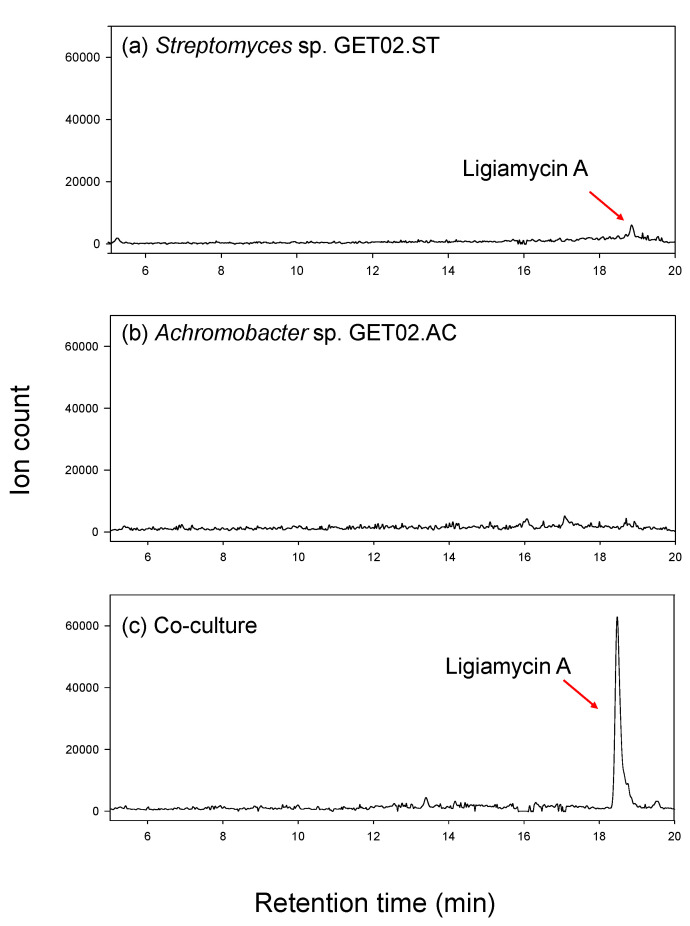
LC/MS traces (ion counts) of ion extraction for the ion [M + H]^+^ at *m*/*z* 317 (ligiamycin A) (**a**) in a pure culture of *Streptomyces* sp. GET02.ST, (**b**) in a pure culture of *Achromobacter* sp. GET02.AC, and (**c**) in a co-culture. The LC/MS analyses were acquired with 10–100% aqueous CH_3_CN over 20 min.

When cultivated in a pure single culture of the strain, *Streptomyces* sp. GET02.ST, isolated from the gut of *Ligia exotica*, produced a distinguished metabolite, ligiamycin A (**1**) with [M + H]^+^ at *m*/*z* 317 and ultraviolet (UV) absorption (*λ*_max_ at 250 and 351 nm). However, the production level was minuscule, and this compound was barely identified by the liquid chromatography (LC)/mass spectrometry (MS) analysis. For further characterization of the compound, we applied the co-culture strategy and enhanced the yield of **1**. Interestingly, when we co-cultured *Streptomyces* sp. GET02.ST with *Achromobacter* sp. GET02.AC, which was also isolated from the gut of *L*. *exotica*, the productivity of **1** was increased by approximately 24 times that of the pure culture of *Streptomyces* sp. GET02.ST (Figure 1). Moreover, an additional derivative of **1**, ligiamycin B (**2**), which was hardly detected in the pure cultured medium of GET02.ST, was identified in the LC/MS profile of the co-cultured (with *Achromobacter* sp. GET02.AC) medium (Figure S18). The significant enhancement of the yields enabled subsequent chromatographic purification and structure elucidation of **1** and **2**.

### 2.2. Structural Elucidation

**Figure 2:**
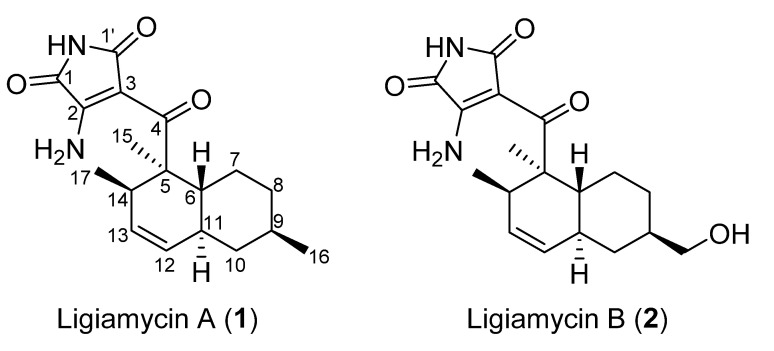
Structures of ligiamycins A (**1**) and B (**2**).

Ligiamycin A (**1**) (Figure 2) was isolated as a white powder, and the molecular formula of compound **1** was deduced to be C_18_H_24_N_2_O_3_, with eight degrees of unsaturation, by the positive high-resolution electrospray ionization mass spectrometry (HR-ESI-MS) analysis ([M + H]^+^ at *m*/*z* 317.1861, calculated as 317.1860) (Figure S15). The ^13^C and multiplicity-edited heteronuclear single quantum coherence spectroscopy (multiplicity-edited HSQC) nuclear magnetic resonance (NMR) spectra of **1** indicated the existence of three carbonyl carbons (*δ*_C_ 200.0, 169.8, and 165.1), two fully substituted olefinic carbons (*δ*_C_ 157.8 and 98.7), two olefinic methine carbons (*δ*_C_ 130.2 and 129.0), eight aliphatic *sp*^3^ carbons (*δ*_C_ 50.1, 42.1, 39.3, 38.0, 35.7, 34.6, 32.9, and 27.2), and three methyl groups (*δ*_C_ 22.5, 18.5, and 14.8). A comprehensive analysis of the ^1^H NMR spectrum in combination with the multiplicity-edited HSQC NMR spectrum showed that **1** possesses two olefinic protons (*δ*_H_ 5.52 and 5.31), four methine protons (*δ*_H_ 3.06, 1.75, 1.55, and 1.48), six methylene protons (*δ*_H_ 1.88, 1.77, 1.70, 1.00, 0.88, and 0.78), three methyl groups (*δ*_H_ 1.27, 0.88, and 0.68), and three heteroatom-bound protons (*δ*_H_ 10.6, 9.16, and 8.90) (Table 1). The two double bonds and three carbonyl groups accounted for five unsaturation degrees, thereby suggesting that ligiamycin A (**1**) contains three rings.

**Figure 3:**
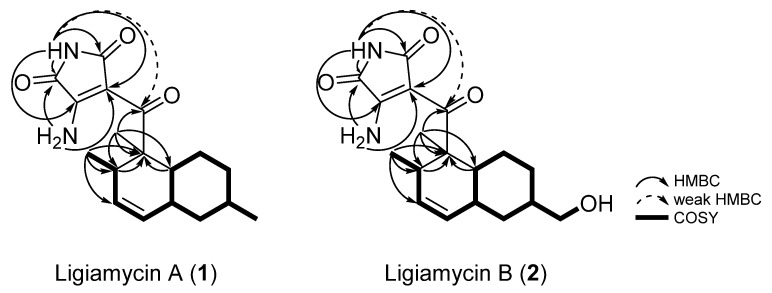
Key HMBC and COSY correlations of ligiamycins A (**1**) and B (**2**).

Comprehensive analyses of ^1^H, ^13^C, and correlation spectroscopy (COSY) NMR spectra was performed to identify partial structures of **1**. The doublet methyl group protons (*δ*_H_ 0.68) at C-17 (*δ*_C_ 18.5) correlated with H-14 (*δ*_H_ 3.06), assigning the C-17 methyl group bound to C-14 (*δ*_C_ 34.6). The H-14/H-13 (*δ*_H_ 5.52) COSY correlation connected C-14 and C-13 (*δ*_C_ 130.2). The 10 Hz coupling constant between H-13 and H-12 (*δ*_H_ 5.31) indicated C-13–C-12 (*δ*_C_ 129.0) connectivity inside a six-membered ring. C-11 (*δ*_C_ 38.0) was then assigned adjacent to C-12 by H-12/H-11 (*δ*_H_ 1.75) COSY coupling. H-11 correlated with H_2_-10 (*δ*_H_ 1.77 and 0.78) and H-6 (*δ*_H_ 1.55), extending the chain to C-10 (*δ*_C_ 42.1) and C-6 (*δ*_C_ 39.3). Furthermore, H-9 (*δ*_H_ 1.48) displayed COSY correlations with H_2_-8 (*δ*_H_ 1.70 and 1.00) and H_3_-16 (*δ*_H_ 0.88), attaching the methylene C-8 (*δ*_C_ 35.7) and the methyl C-16 (*δ*_C_ 22.5) groups to C-9 (*δ*_C_ 32.9). A H_2_-8/H_2_-7 (*δ*_H_ 1.88 and 0.88) COSY correlation located C-7 (*δ*_C_ 27.2) next to C-8. H-6, which showed a COSY correlation with H-11, as mentioned, also correlated with H_2_-7, constructing a methylcyclohexane moiety. This substructure accounted for one of the predicted three rings. The other six-membered ring, which was deduced by ^3^*J*_H12H13_ (10 Hz), was elucidated as a dimethylcyclohexene (C-6, C-7, C-11, C-12 (*δ*_C_ 129.0), C-13 (*δ*_C_ 130.2), and C-14 with the two methyl groups C-15 and C-17) based on the HMBC correlations from the singlet methyl protons H_3_-15 (*δ*_H_ 1.27) to C-6, C-5 (*δ*_C_ 50.1), and C-14 and from H-6 to C-5 and H-14 to C-5. This connectivity was also confirmed by the H_3_-17/C-5 heteronuclear coupling. Thus, the two identified six-membered rings were coupled as a decalin structure (Figure 3).

As the elucidated decalin moiety was composed of C_13_H_21_, C_5_H_3_N_2_O_3_ remained to construct the full structure of ligiamycin A (**1**). An analysis of the ^1^H-^15^N HSQC NMR spectrum identified that all three exchangeable protons were bound at nitrogen. Among them, two protons, 2-NH_2a_ (*δ*_H_ 9.16) and 2-NH_2b_ (*δ*_H_ 8.90), were connected to the same nitrogen at *δ*_N_ 103.8, forming a primary amine group (Figure S8). On the contrary, the remaining proton (1,1’-NH) at *δ*_H_ 10.60 was revealed as an amide proton based on the chemical shift (*δ*_N_ 146.8) of its 1-bond nitrogen and HMBC correlations from 1,1’-NH to two carbonyl carbons (C-1 and C-1’; *δ*_C_ 165.1 and 169.8).

Based on HMBC correlations from 1,1’-NH (*δ*_H_ 10.60) to C-2 (*δ*_C_ 157.8) and C-3 (*δ*_C_ 98.7) and from 2-NH_2_ to C-1 and C-3, the last remaining ring structure was elucidated as 3-amino-maleimide (3-amino-1*H*-pyrrole-2,5-dione). The established two substructures, decalin and 3-amino-1*H*-pyrrole-2,5-dione, were assembled through C-4 (*δ*_C_ 200.0) based on a HMBC correlation from H_3_-15 to C-4 and a weak HMBC correlation from 1,1’-NH to C-4, determining the full planar structure of ligiamycin A (**1**) (Figure 3).

Ligiamycin B (**2**) (Figure 1) was isolated as a white powder, and its molecular formula was deduced as C_18_H_24_N_2_O_4_ having eight degrees of unsaturation based on the positive high-resolution fast atom bombardment mass spectrometry (HR-FAB-MS) analysis ([M + H]^+^ at *m*/*z* 333.1816, calculated as 333.1814) (Figure S16). Interpretation of the HSQC NMR spectrum allowed for all one-bond ^1^H-^13^C assignments. By comparing the 1D and 2D NMR spectra of **1** and **2**, ligiamycin B (**2**) has one less doublet methyl group than ligiamycin A (**1**), but instead possesses one heteroatom-bound methylene group (*δ*_H_ 3.22/*δ*_C_ 66.5) and one exchangeable proton at *δ*_H_ 4.36. An analysis of the COSY NMR spectrum of **2** revealed that this proton (*δ*_H_ 4.36) was bound to the oxygen atom attached to C-16 based on the COSY signal of H_2_-16 (*δ*_H_ 3.22)/16-OH (Figure 3). The substitution of this hydroxy group affected the ^13^C chemical shifts. Compared to the ^13^C chemical shifts of **1**, C-9 (*δ*_C_ 40.9) resonated in a lower field by the *β*-effect, whereas C-8 (*δ*_C_ 30.3) and C-10 (*δ*_C_ 36.8) appeared in a higher field by the *γ*-effect of this substitution. The full structure of **2** was elucidated as a C-16 hydroxy analogue of **1** by COSY and HMBC correlations (Figure 3).

**Figure 4:**
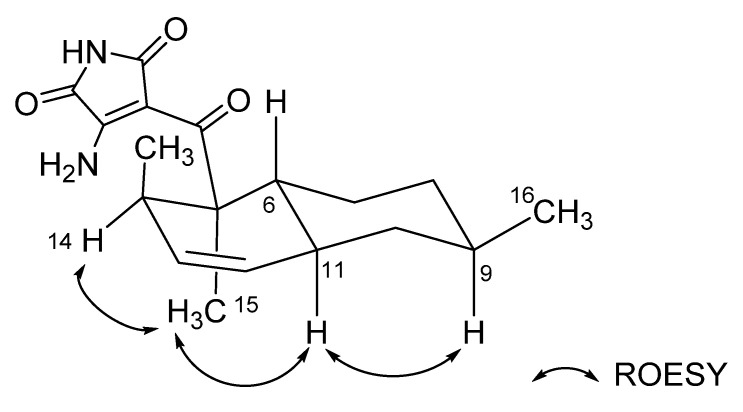
Key ROESY correlations of ligiamycin A (**1**).

The relative configurations of the decalin structure in ligiamycin A (**1**) were determined by an analysis of the rotating frame Overhauser effect spectroscopy (ROESY) NMR spectrum (Figures S6 and S7). H-9/H-11, H-11/H_3_-15, and H_3_-15/H-14 ROESY correlations indicated that H-9, H-11, H-14, and H_3_-15 are oriented to the same phase, which elucidated 5R*, 6R*, 9R*, 11S*, and 14R* configurations along with *trans*-decalin conformation for **1** (Figure 4 and Figure S17a). The relative configurations of ligiamycin B (**2**) were also established as 5R*, 6R*, 9R*, 11S*, and 14R* by its ROESY correlations, analogous to those of **1** (Figure S17b).

For the purpose of deducing the absolute configurations of the ligiamycins, we attempted electronic circular dichroism (ECD) calculations [16]. Energy-minimized structures of the two possible enantiomers (5*R*/6*R*/9*R*/11*S*/14*R* and 5*S*/6*S*/9*S*/11*R/*14*S*) of **1** and **2** were constructed, and their ECD calculations were performed. However, the experimental ECD spectra of **1** and **2** did not display consistency with any calculated ECD spectra of **1** and **2** (Figures S19 and S20). Then, we conducted optical rotation calculations [17,18,19,20]. The calculated value of optical rotation of the 5*R*, 6*R*, 9*R*, 11*S*, and 15*R* enantiomer had a negative optical rotation value of ${\left[\alpha \right]}_{D}^{25}$ − 65, whereas a positive optical rotation value (${\left[\alpha \right]}_{D}^{25}$ + 66) was calculated for the opposite enantiomer. Based on the measured optical rotation value of **1** (${\left[\alpha \right]}_{D}^{25}$ − 78), the absolute configurations of **1** were deduced as 5*R*, 6*R*, 9*R*, 11*S*, and 14*R* (Table 2). In the same manner, the absolute configurations of ligiamycin B (**2**) were inferred as 5*R*, 6*R*, 9*R*, 11*S*, and 14*R* (Table 2).

### 2.3. Biological Evaluation

We evaluated the antibacterial (*Staphylococcus aureus* ATCC25923, *Enterococcus faecalis* ATCC19433, *Enterococcus faecium* ATCC19434, *Klebsiella pneumoniae* ATCC10031, *Salmonella enterica* ATCC14028, and *Escherichia coli* ATCC25922) and antifungal (*Aspergillus fumigatus* HIC6094, *Trichophyton rubrum* NBRC9185, *Trichophyton mentagrophytes* IFM40996, and *Candida albicans* ATCC10231) activities of ligiamycins. Ligiamycin A (**1**) showed moderate inhibitory effects against *S. aureus* and *S. enterica*, whereas ligiamycin B (**2**) exhibited weak or no inhibitory effects when using ampicillin and tetracycline as positive control compounds, implying that the presence of a hydroxy group in **2** might play a negative role in antibacterial effects. Both ligiamycins A (**1**) and B (**2**) did not show any significant inhibitory effects (MIC > 128 μg/mL) against the tested fungal strains (*C*. *albicans*, *A*. *fumigatus*, *T*. *rubrum*, and *T. mentagrophytes*) when using amphotericin B as a positive control compound (Table 3).

In our cell proliferation assay against the human carcinoma cell lines SNU638 (human gastric cancer cells), SK-HEP-1 (human liver cancer cells), A549 (human lung cancer cells), HCT116 (human colorectal cancer cells), and MDA-MB-231 (human breast cancer cells), ligiamycin A (**1**) did not show cytotoxicity, whereas ligiamycin B (**2**), which bears a hydroxy group (16-OH), displayed moderate cell cytotoxicity against HCT116 cancer cells (20.1 μM) (Table 4).

## 3. Materials and Methods

### 3.1. General Experimental Procedure

Optical rotations of the ligiamycins were acquired using a JASCO P-2000 polarimeter (sodium light source, JASCO, Easton, PA, USA) with a 1 cm cell. CD and UV spectra were obtained by using Applied Photophysics Ltd., Chirascan Plus (Applied Photophysics, Leatherhead, Surrey, UK). Infrared (IR) spectra were obtained using JASCO, FT/IR-4200 (Thermo, Madison, CT, USA). All of the LC/MS data were collected by an Agilent Technologies 1200 series HPLC instrument (Agilent Technologies, Santa Clara, CA, USA) tandemly coupled with an Agilent Technologies 6130 quadrupole MS (Agilent Technologies, Santa Clara, CA, USA) equipped with an ESI source. 1D and 2D NMR spectra were acquired on Bruker Avance 800 and 850 MHz NMR spectrometers (Bruker, Billerica, MA, USA) located at the College of Pharmacy, Seoul National University and the National Center for Inter-University Research Facilities (NCIRF), Seoul National University, respectively. HR-ESI-MS for ligiamycin A (**1**) was collected by a high-resolution LC/MSMS spectrometer (Q-TOF 5600, AB SCIEX, Framingham, MA, USA) at the National Instrumentation Center for Environment Management (NICEM) in the College of Agriculture and Life Sciences, Seoul National University. HR-FAB-MS for ligiamycin B (**2**) was obtained using a gas chromatography high-resolution mass spectrometer (JMS-700, 6890 Series, JEOL, Akishima, Tokyo, Japan) at NCIRF, Seoul National University.

### 3.2. Collection and Bacterial Isolation of Wharf Roaches

The wharf roach specimens belonging to the marine isopod were collected at the mudflat experience center (36°8’19.02”, 126°34’58.0”) in Seocheon, Chungcheongnam-do, Korea, in April 2018. The specimens were morphologically identified as *Ligia exotica* [12]. The outer skins of the wharf roach specimens were sterilized by fresh ethanol (EtOH). The gut samples of the wharf roaches were isolated using a sterilized razor blade and suspended in 40 mL of sterilized distilled water. For the purpose of bacterial isolation from the gut of the wharf roaches, 400 µL of the suspension was dropped and spread on various isolation agar media (Table S1). GET02.ST and GET02.AC strains were isolated on modified K medium. The GET02.ST strain was identified as *Streptomyces* sp. (GenBank accession number: MZ675370), which was closest to *Streptomyces* sp. 4K301 (95% identity; GenBank accession number: MG770872.1). The GET02.AC strain was identified as *Achromobacter* sp. (GenBank accession number: MZ675369), which was most closely related to *Achromobacter* sp. CLC-M23 (99% identity; GenBank accession number: MH518245.1).

### 3.3. Large-Scale Co-Culture and Extraction

Both the GET02.ST and GET02.AC strains were cultivated in 50 mL of modified K medium (3 g of yeast extract, 2 g of glucose, 2 g of mannitol, 5 g of malt extract, 5 g of soluble starch, 5 g of soytone, 1 g of calcium carbonate, and 23 g of sea salt in 1 L of distilled water) in a 125 mL seed flask. After fermentation for 3 days on a rotary shaker at 170 rpm and 30 °C, 10 mL of the seed culture was inoculated in 250 mL of modified K medium in a 500 mL flask. After culturing for 4 days on a rotary shaker at 170 rpm and 30 °C, 7 mL of the GET02.ST strain and 3 mL of the GET02.AC strain were transferred together into 250 mL of modified K medium (48 × 250 mL; total volume of 12 L) for co-culture and then cultured for 8 days on a rotary shaker at 170 rpm and 30 °C. A total volume of 12 L of the whole co-culture was extracted with 27 L of ethyl acetate (EtOAc). The EtOAc and water layers were separated, and the residual water in the EtOAc layer was eliminated by adding anhydrous Na_2_SO_4_. The extract was concentrated in a rotary evaporator, obtaining 2 g of dry material.

### 3.4. Isolation and Purification of Ligiamycins

The crude extract was re-dissolved by methanol (MeOH) and then filtered by a 25HP045AN (ADVANTEC, Tokyo, Japan) syringe filter. The extract was directly injected into a reversed-phase high-performance liquid chromatography (HPLC) column (Phenomex Luna C_18_(2), 4.6 × 250 mm) with a step gradient solvent system (30–40% CH_3_CN-H_2_O gradient solvent system for 40 min; after 40 min, 60% CH_3_CN-H_2_O isocratic solvent system, flow rate 1 mL/min, and UV detection at 254 nm). Ligiamycins A (**1**) and B (**2**) were eluted at 59 and 29 min, respectively. **1** was further purified with the gradient solvent system (30–40% CH_3_CN-H_2_O gradient solvent system for 40 min, flow rate 1 mL/min, and UV detection at 254 nm) with the same reversed-phase HPLC column. Purified **1** (4 mg) was eluted at 37 min. **2** was also further chromatographed for purification with a gradient solvent system (55–77% CH_3_CN-H_2_O gradient solvent system for 40 min, flow rate 1 mL/min, and UV detection at 254 nm) with the same reversed-phase HPLC column. Purified **2** (2 mg) was eluted at 29 min.

#### 3.4.1. Ligiamycin A (**1**)

White powder; ${\left[\alpha \right]}_{D}^{25}$ − 78 (c = 0.1, MeOH); UV(MeOH) *λ*_max_ (log *ε*) 250 (3.80), 351 (2.31) nm; IR (neat) *ν*_max_ 3387, 2959, 1729, 1648, 1516, 1463, 1369, and 1055 cm^−1^; for ^1^H and ^13^C NMR spectral data, Table 1; HR-ESI-MS [M + H]^+^ *m*/*z* 317.18615 (calcd. for C_18_H_25_N_2_O_3_, 317.18597).

#### 3.4.2. Ligiamycin B (**2**)

White powder; ${\left[\alpha \right]}_{D}^{25}$ − 67 (c = 0.1, MeOH); UV(MeOH) *λ*_max_ (log *ε*) 250 (3.38), 352 (1.92) nm; IR (neat) *ν*_max_ 3386, 2923, 1724, 1651, 1515, 1463, 1356, and 1036 cm^−1^; for ^1^H and ^13^C NMR spectral data, Table 1; HR-FAB-MS [M + H]^+^ *m*/*z* 333.1816 (calcd. for C_18_H_25_N_2_O_4_, 333.1814).

### 3.5. ECD and Optical Rotation Calculation

The ECD calculation was performed as described previously [20]. The possible conformers of ligiamycins A (**1**) and B (**2**) were obtained based on the detailed analysis of ROESY spectroscopic data, and the energy-minimized conformational structures were calculated by Avogadro 1.2.0 [21]. All DFT calculations for ground state geometries of the ligiamycins were acquired by Turbomole 4.3.2. (TmoleX) [22] with the def-SVP set in all atoms at the density functional theory (DFT) (Table S2). ECD data of ligiamycins were obtained by overlapping each transition, where *σ* is the width of the band at 1/*e* height. *ΔE_i_* and *R_i_* are the rotary strengths and excitation energies for transition *i*, respectively. For this study, *σ* was fixed at 0.10 Ev (Table S3). The calculated optical rotations of **1** and **2** corresponding to the optimized structures were obtained by TmoleX using DFT with functional B3-LYP and the basis set of def-SV(P) via TmoleX.

$$\Delta \epsilon \left(E\right)=\frac{1}{2.297\times 10^{-39}}\frac{1}{\sqrt{2\pi \sigma }}\sum_{i}^{A}\Delta E_{i}R_{i}e^{[-{\left(E-\Delta E_{i}\right)}^{2}/\left(2\sigma {)}^{2}\right]}$$

### 3.6. Cell Culture

All the cancer cell lines (SNU-638, SK-HEP-1, A549, HCT116, and MDA-MB-231 cancer cells) were obtained from the American Type Culture Collection (Manassas, VA, USA). SNU-638, A549, and HCT116 cells were cultured in Roswell Park Memorial Institute 1640 medium, and SK-HEP-1 and MDA-MB-231 cells were cultured in Dulbecco’s Modified Eagle’s medium. All media were supplemented with a penicillin–streptomycin mixture (10,000 units/mL sodium penicillin G and 10,000 μg/mL streptomycin) and 10% fetal bovine serum. They were incubated in a humidified incubator that contained 5% CO_2_ at 37 °C.

### 3.7. Cell Proliferation Assays

A sulforhodamine B (SRB) assay was performed for cell proliferation as described previously [23]. Cancer cells were seeded in 96-well plates and then incubated for 72 h with treatments of the ligiamycins. After 72 h, 10% trichloroacetic acid was used to fix the cells (30 min) and then washed with deionized water and dried overnight. Proteins were stained using a 0.4% SRB solution in 1% acetic acid; unbound dye was cleared using 1% acetic acid. After dissolving unbound dye with 10 mM Tris buffer (pH = 10.0), the absorbance of the cell solution was measured by a Versamax ELISA instrument (515 nm, Molecular Devices, LLC, San Jose, CA, USA). TableCurve 2D v5.01 software (Systant Software Inc., Richmond, CA, USA) was utilized to determine the IC_50_ values.

### 3.8. Antibacterial Activity Bioassays

The inhibitory activities of ligiamycins were tested against Gram-positive and Gram-negative bacteria in a similar way as described previously [24,25]. Gram-positive and Gram-negative bacteria were cultured in Mueller Hinton Broth (MHB) overnight. The cells were centrifuged and washed twice with sterilized distilled water. Ligiamycins A (**1**) and B (**2**) were dissolved in dimethyl sulfoxide (DMSO) separately and diluted with MHB for the purpose of preparing serial twofold dilutions in the range of 0.06–128 μg/mL. In each well of a 96-well plate, 190 μL of MHB containing the test compound (each **1** and **2**) was mixed with 10 μL of broth containing the test bacterium (final concentration: 5 × 10^5^ colony-forming units (cfu)/mL) adjusted to match the turbidity of a 0.5 MacFarland standard. The plates were incubated at 37 °C for 24 h. The MIC values were decided as the lowest concentration of the test compound that prevented cell growth. Ampicillin and tetracycline were used as control compounds.

## 4. Conclusions

The co-cultivation of marine wharf roach gut bacterial strains *Streptomyces* sp. GET02.ST and *Achromobacter* sp. GET02.AC induced the production of secondary metabolites from *Streptomyces* sp. GET02.ST by 24 times, leading to the discovery and structure determination of two new natural products, ligiamycins A (**1**) and B (**2**). The configurations of **1** and **2** were established by analyses of ROESY correlations and calculated optical rotations. Ligiamycins A (**1**) and B (**2**) displayed a unique structural feature by coupling a decalin with an amino maleimide moiety. Oxasetin, which was discovered from the fungus *Vaginatispora aquatica*, is structurally the most similar by bearing decalin and maleimide [26]. Dysidinoid A and the oxaleimides also have related structures to decalin and maleimide [27,28]. However, these compounds have hydroxy maleimide or different substitution patterns to decalin. The amino-maleimide substructure is extremely rare in nature. The only previously reported natural products with this moiety are hyperectine and isohyperectine from the plants *Hypecoum erectum* and *Hypecoum leptocapum* [29,30,31]. Therefore, ligiamycins A (**1**) and B (**2**) are the first amino-maleimide-bearing compounds from microorganisms. In addition, thus far, the combinations of decalin and maleimide have been reported only from fungi, highlighting that ligiamycins A (**1**) and B (**2**) are the first bacterial decalin–maleimide metabolites. Based on the structural similarity to oxaleimides, we proposed that ligiamycins were putatively biosynthesized through a polyketide synthase–nonribosomal peptide synthetase (PKS-NRPSs) hybrid pathway (Scheme S1) [28]. Ligiamycins A (**1**) and B (**2**) are biologically active with antibacterial activity and a weak cytotoxic effect against the tested cancer cell lines. It is still unclear how *Achromobacter* sp. GET02.AC elicits the production of the ligiamycins from *Streptomyces* sp. GET02.ST. Based on the previous reports regarding other co-cultures [32], cell–cell contact [4] or small molecule inducers [33] could be the eliciting mechanisms, but elucidating the role of *Achromobacter* sp. GET02.AC in this co-culture requires further comprehensive studies. Our finding about the ligiamycins demonstrates that the co-cultivation of ecologically relevant microbes enhances the production of otherwise neglected minor but structurally unique metabolites and provides an effective approach to discovering new bioactive compounds. Furthermore, utilizing the gut microflora of chemically under-investigated wharf roaches for exploring new chemical entities indicates that unexplored marine invertebrates such as wharf roaches could serve as sources to expand microbial chemical diversity.

## Figures and Tables

**Table 1:** ^1^H, ^13^C, and ^15^N NMR data for ligiamycins A (**1**) and B (**2**) in DMSO-*d*_6_.

	Ligiamycin A (1) ^a^		Ligiamycin B (2) ^b^	
Position	*δ*_C_/*δ*_N_, Type	*δ*_H_, Mult (*J* in Hz)	*δ*_C_, Type	*δ*_H_, Mult (*J* in Hz)
1	165.1, C		165.2, C	
1’	169.8, C		169.9, C	
2	157.8, C		157.8, C	
3	98.7, C		98.7, C	
4	200.0, C		200.0, C	
5	50.1, C		50.1, C	
6	39.3, CH	1.55, t (10.0)	39.6, CH	1.56, t (10.0)
7a	27.2, CH_2_	1.88, m	26.8, CH_2_	1.92, m
7b		0.88, m ^c^		0.86, m
8a	35.7, CH_2_	1.70, m	30.3, CH_2_	1.75, m
8b		1.00, ddd (16.0, 12.5, 3.5)		0.99, m
9	32.9, CH	1.48, m	40.9, CH	1.47, m
10a	42.1, CH_2_	1.77, m	36.8, CH_2_	1.84, m
10b		0.78, m		0.76, m
11	38.0, CH	1.75, m	37.7, CH_2_	1.73, m
12	129.0, CH	5.31, dd (10.0, 2.0)	129.1, CH	5.33, dd (10.0, 2.0)
13	130.2, CH	5.52, m	130.2, CH	5.52, m
14	34.6, CH	3.06, m	34.6, CH	3.06, m
15	14.8, CH_3_	1.27, s	14.8, CH_3_	1.28, s
16	22.5, CH_3_	0.88, m ^c^	66.5, CH_2_	3.22, d (6.0)
17	18.5, CH_3_	0.68, d (7.0)	18.5, CH_3_	0.68, d (6.5)
1,1’-NH	146.8 ^d^, NH	10.6, br s		10.6, br s
2-NH_2a_	103.9 ^d^, NH_2_	9.16, s		9.16, s
2-NH_2b_		8.90, br s		8.89, br s
16-OH				4.36, br s

^a 1^H and ^13^C NMR data were recorded at 800 and 200 MHz, respectively. ^b 1^H and ^13^C NMR data were recorded at 850 and 225.5 MHz, respectively. ^c^ Overlapping signals. ^d^ The chemical shifts of ^15^N in the groups of N were determined based on the ^1^H-^15^N HSQC NMR spectrum.

**Table 2:** Experimental and calculated optical rotation of ligiamycins A (**1**) and B (**2**).

Sample	Optical Rotation		
	Experimental	Calculated of 5*R*, 6*R*, 9*R*, 11*S*, 14*R*	Calculated of 5*S*, 6*S*, 9*S*, 11*R*, 14*S*
Ligiamycin A (1)	−78	−65	+66
Ligiamycin B (2)	−67	−64	+64

**Table 3:** Inhibitory effects of ligiamycins against bacterial strains.

Sample	MIC (μg/mL)					
	Gram-Positive			Gram-Negative		
	*S. aureus*	*E. faecalis*	*E. faecium*	*K. pneumonia*	*S. enterica*	*E. coli*
Ligiamycin A (1)	16	>128	>128	>128	16	>128
Ligiamycin B (2)	64	>128	>128	>128	>128	>128
Ampicillin	0.13	0.25	0.5	128	0.28	32
Tetracycline	0.13	0.25	0.13	0.5	0.25	0.5

**Table 4:** Inhibitory effects of ligiamycins on the proliferation of human cancer cell lines.

IC_50_ (μM)	SNU638	SK-HEP-1	A549	HCT116	MDA-MB-23
Ligiamycin A (1)	>50	>50	42.0	>50	>50
Ligiamycin B (2)	46.2	34.6	25.9	20.1	23.7
Etoposide	2.17	0.24	0.35	0.66	2.64

## Data Availability

All data are contained within this article and Supplementary Materials.

## Supplementary Materials

The following are available online at https://www.mdpi.com/article/10.3390/md20020083/s1, Figures S1–S14: NMR spectra of ligiamycins; Figure S15: HR-ESI-MS data of ligiamycin A (**1**); Figure S16: HR-FAB-MS data of ligiamycin B (**2**); Figure S17: Energy-minimized conformations of (a) ligiamycin A (**1**) and (b) ligiamycin B (**2**); Figure S18: LC/MS traces (ion counts) of ion extraction for the ion [M-H]^−^ at *m*/*z* 331 (ligiamycin B); Figures S19 and S20: Comparison of the experimental ECD data of ligiamycins with the calculated ECD data; Table S1: Composition of isolation agar media; Table S2: Cartesian coordinates of ligiamycins A (**1**) and B (**2**); Table S3: ECD calculations of ligiamycins A (**1**) and B (**2**); Scheme S1: Proposed biosynthetic pathway of the ligiamycins.
