# Supplementary material for: Ligiamycins A and B, Decalin-Amino-Maleimides from the Co-Culture of Streptomyces sp. and Achromobacter sp. Isolated from the Marine Wharf Roach, Ligia exotica
Source: Mar Drugs. 2022 Jan 18;20(2):83. doi: 10.3390/md20020083 (PMC8878407; doi:10.3390/md20020083)
Supplement: Supplementary file 1 [file marinedrugs-20-00083-s001.zip › marinedrugs-1519459-supplementary.pdf]

Supplementary Material for

Ligiamycins A and B, Decalin-Amino-Maleimides from Co-culture of *Streptomyces* sp. and *Achromobacter* sp. isolated from the Marine Wharf Roach, *Ligia exotica*

Hyung-Ju Lim [1,†], Joon Soo An [1,†], Eun Seo Bae [1], Eunji Cho [2], Sunghoon Hwang [1], Sang-Jip Nam [3], Ki-Bong Oh [2],Sang Kook Lee [1] and Dong-Chan Oh [1,*]

[1]Natural Products Research Institute, College of Pharmacy, Seoul National University, Seoul 08826, Republic of Korea; limju012@snu.ac.kr (H.-J.L.); ahnjunsoo@snu.ac.kr (J.S.A.); ddol1289@snu.ac.kr (E.S.B); sunghooi@snu.ac.kr (S.H.); sklee61@snu.ac.kr (S.K.L.)

[2]Department of Agriculture Biotechnology, College of Agriculture and Life Sciences, Seoul National University, Seoul 08826, Republic of Korea; eunji525@snu.ac.kr (E.C.); ohkibong@snu.ac.kr (K.-B.O.)

[3]Department of Chemistry and Nanoscience, Ewha Womans University, Seoul 03760, Republic of Korea; sjnam@ewha.ac.kr (S.-J.N.)

[†]These authors contributed equally to this work.

Corresponding Author

* D.-C. Oh, Tel: +82-2-880-2491, Fax: +82-2-762-8322, E-mail: dongchanoh@snu.ac.kr.

Table of Contents

Figure S1. ^{1}H NMR spectrum (800 MHz) of ligiamycin A (**1**) in DMSO-d_6.
Figure S2. ^{13}C NMR spectrum (200 MHz) of ligiamycin A (**1**) in DMSO-d_6.
Figure S3. COSY NMR spectrum (800 MHz) of ligiamycin A (**1**) in DMSO-d_6.
Figure S4. HSQC NMR spectrum (800 MHz) of ligiamycin A (**1**) in DMSO-d_6.
Figure S5. HMBC NMR spectrum (800 MHz) of ligiamycin A (**1**) in DMSO-d_6.
Figure S6. ROESY NMR spectrum (800 MHz) of ligiamycin A (**1**) in DMSO-d_6.
Figure S7. Expanded ROESY NMR spectrum (800 MHz) of ligiamycin A (**1**) in DMSO-d_6.
Figure S8. ^{1}H-^{15}N HSQC NMR spectrum (850 MHz) of ligiamycin A (**1**) in DMSO-d_6.
Figure S9. ^{1}H NMR spectrum (800 MHz) of ligiamycin B (**2**) in DMSO-d_6.
Figure S10. ^{13}C NMR spectrum (200 MHz) of ligiamycin B (**2**) in DMSO-d_6.
Figure S11. COSY NMR spectrum (800 MHz) of ligiamycin B (**2**) in DMSO-d_6.
Figure S12. HSQC NMR spectrum (800 MHz) of ligiamycin B (**2**) in DMSO-d_6.
Figure S13. HMBC NMR spectrum (800 MHz) of ligiamycin B (**2**) in DMSO-d_6.
Figure S14. ROESY NMR spectrum (800 MHz) of ligiamycin B (**2**) in DMSO-d_6.
Figure S15. HR-ESI-MS data of ligiamycin A (**1**).
Figure S16. HR-FAB-MS data of ligiamycin B (**2**).
Table S1. Composition of isolation agar media.
Figure S17. Energy-minimized conformations of (a) ligiamycin A (**1**) and (b) ligiamycin B (**2**).
Figure S18. LC/MS traces (ion counts) of ion extraction for the ion $[M-H]^-$ at *m/z* 331 (ligiamycin B) (a) in a pure culture of *Streptomyces* sp. GET02.ST, (b) in a pure culture of *Achromobacter* sp. GET02.AC, and (c) in a co-culture. The LC/MS analyses were acquired with 10%–100% aqueous CH_3CN over 20 min.
Table S2. Cartesian coordinates of ligiamycins A (**1**) and B (**2**).
Table S3. ECD calculations of ligiamycins A (**1**) and B (**2**).
Figure S19. Comparison of the experimental ECD data of ligiamycin A (**1**) with the calculated ECD data; black line: experimental ECD data; blue dotted line: calculated ECD data (5*S*, 6*S*, 9*S*, 11*R*, and 14*S*); red dotted line: calculated ECD data (5*R*, 6*R*, 9*R*, 11*S*, and 14*R*).
Figure S20. Comparison of the experimental ECD data of ligiamycin B (**2**) with the calculated ECD data; black line: experimental ECD data; blue dotted line: calculated ECD data (5*S*, 6*S*, 9*S*, 11*R*, 14*S*); red dotted line: calculated ECD data (5*R*, 6*R*, 9*R*, 11*S*, 14*R*).
Proposed biosynthetic pathway of ligiamycins A (1) and B (2).
Scheme S1. Proposed biosynthetic pathway of ligiamycins. PKS-NRPS domain abbreviations: KS: ketosynthase; DH: dehydratse; MT: methyltransferase; KR: ketoreductases; ACP: acyl carrier protein; C: condensation; A: adenylation; T: thiolation; R: reductase; ER: enoylreductase.
Reference

Figure S1. ^{1}H NMR spectrum (850 MHz) of ligiamycin A (**1**) in DMSO-d_6.

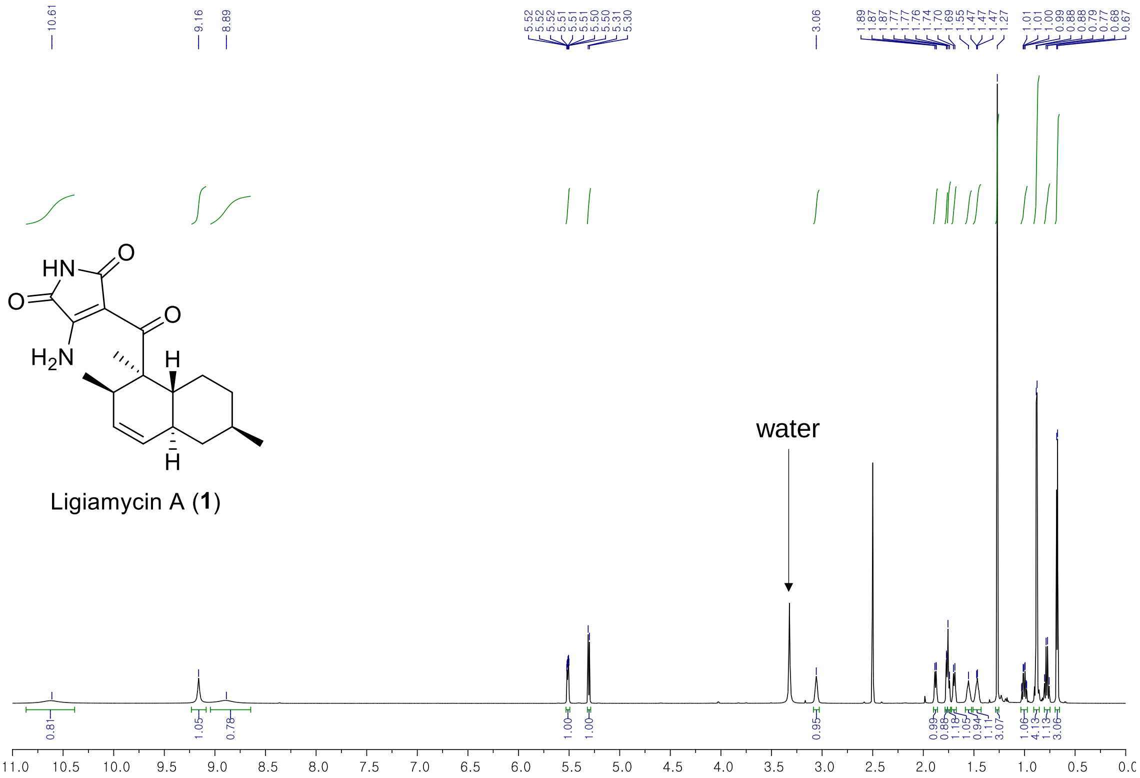

Figure S2. ^{13}C NMR spectrum (215.5 MHz) of ligiamycin A (**1**) in DMSO-d_6.

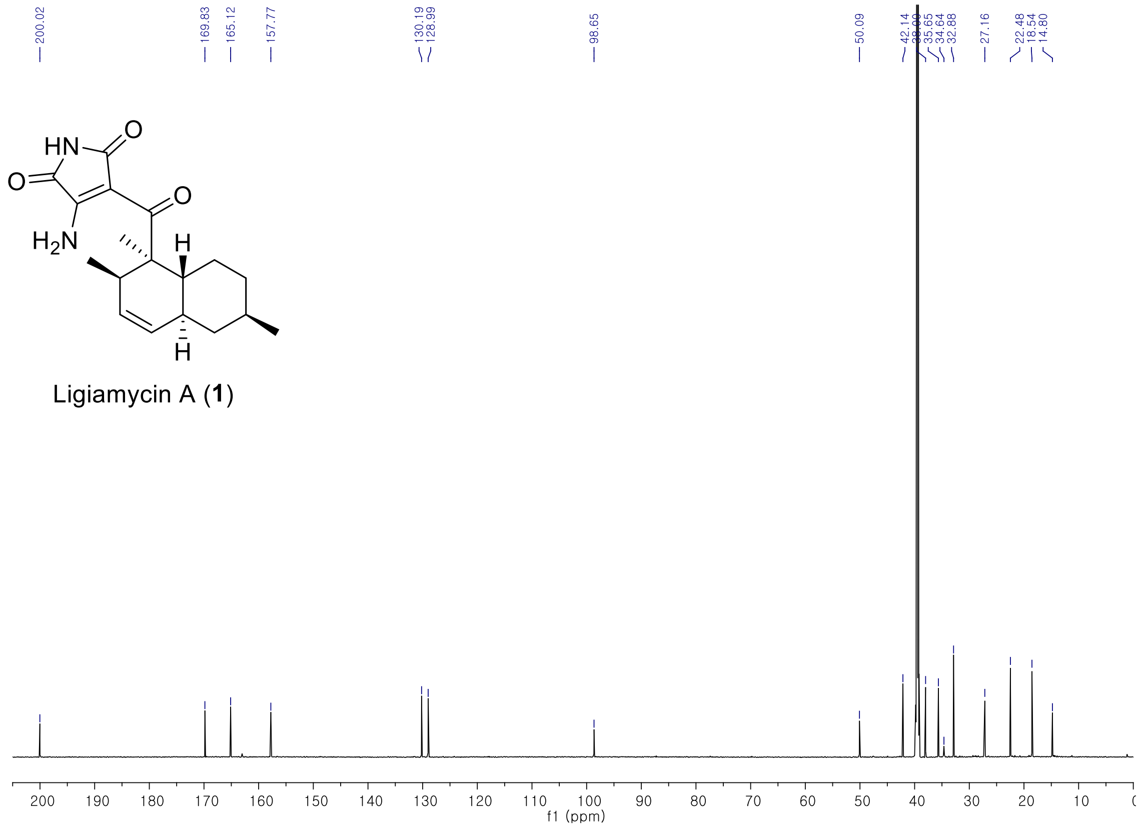

Figure S3. COSY NMR spectrum (800 MHz) of ligiamycin A (**1**) in DMSO-d_6.

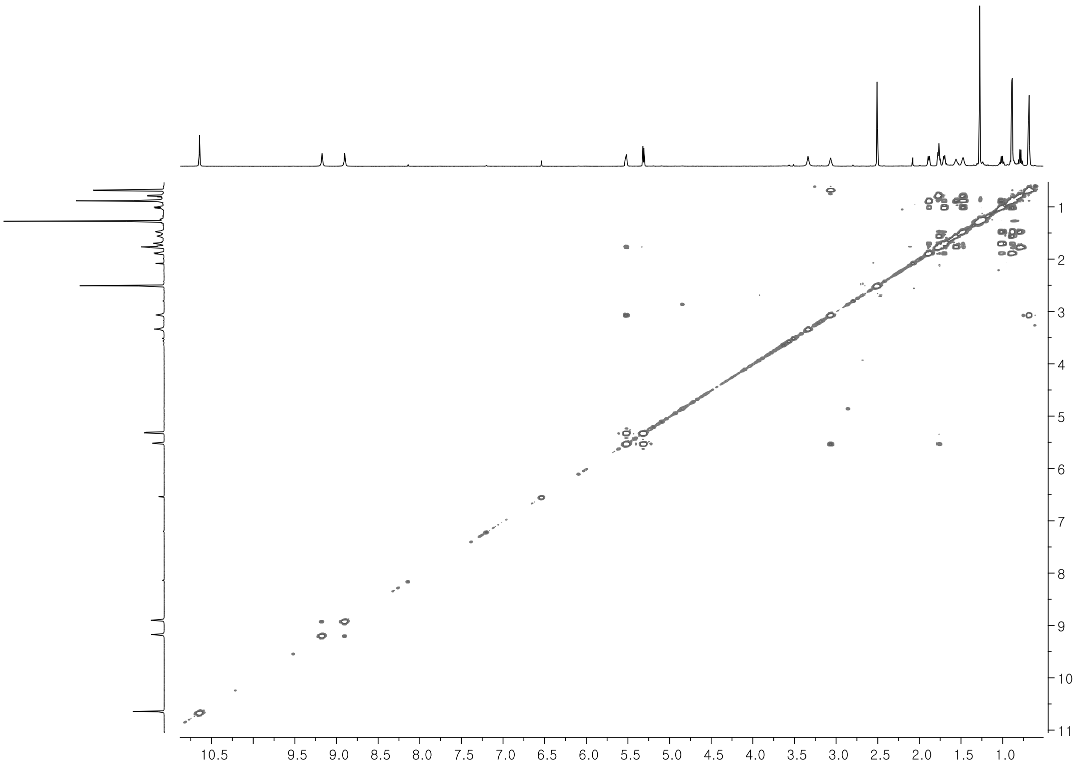

Figure S4. HSQC NMR spectrum (800 MHz) of ligiamycin A (**1**) in DMSO-d_6.

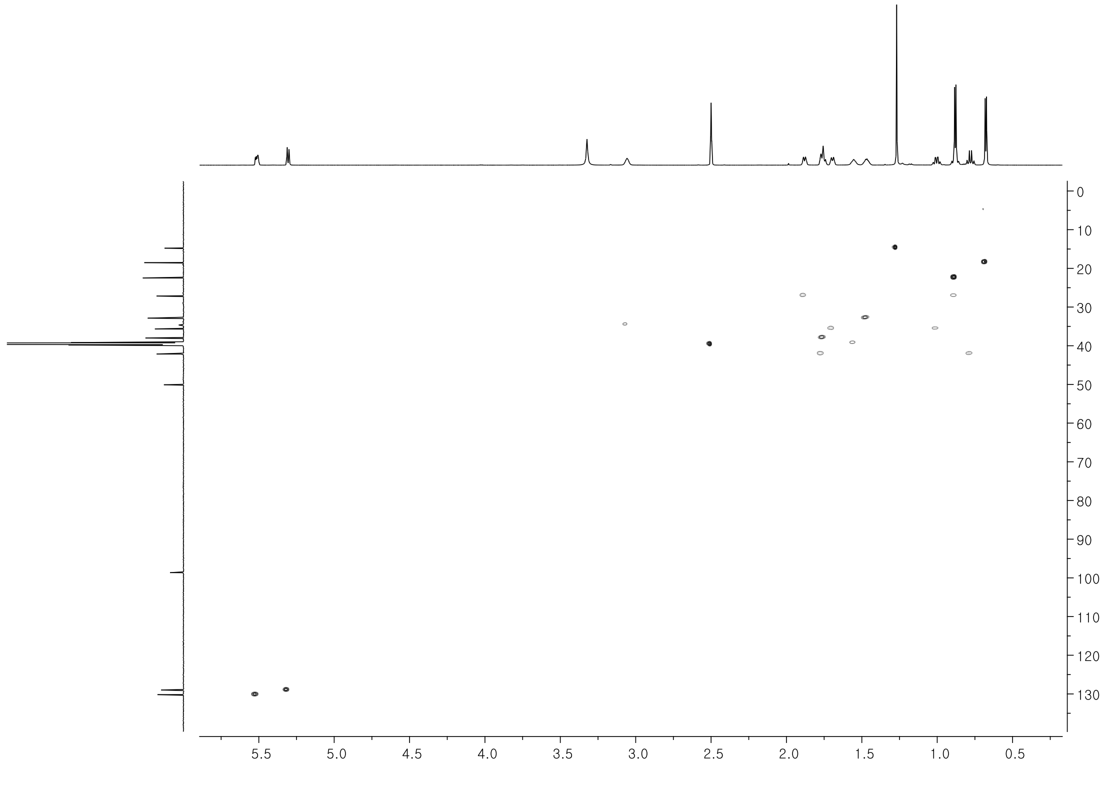

Figure S5. HMBC NMR spectrum (800 MHz) of ligiamycin A (**1**) in DMSO-d_6.

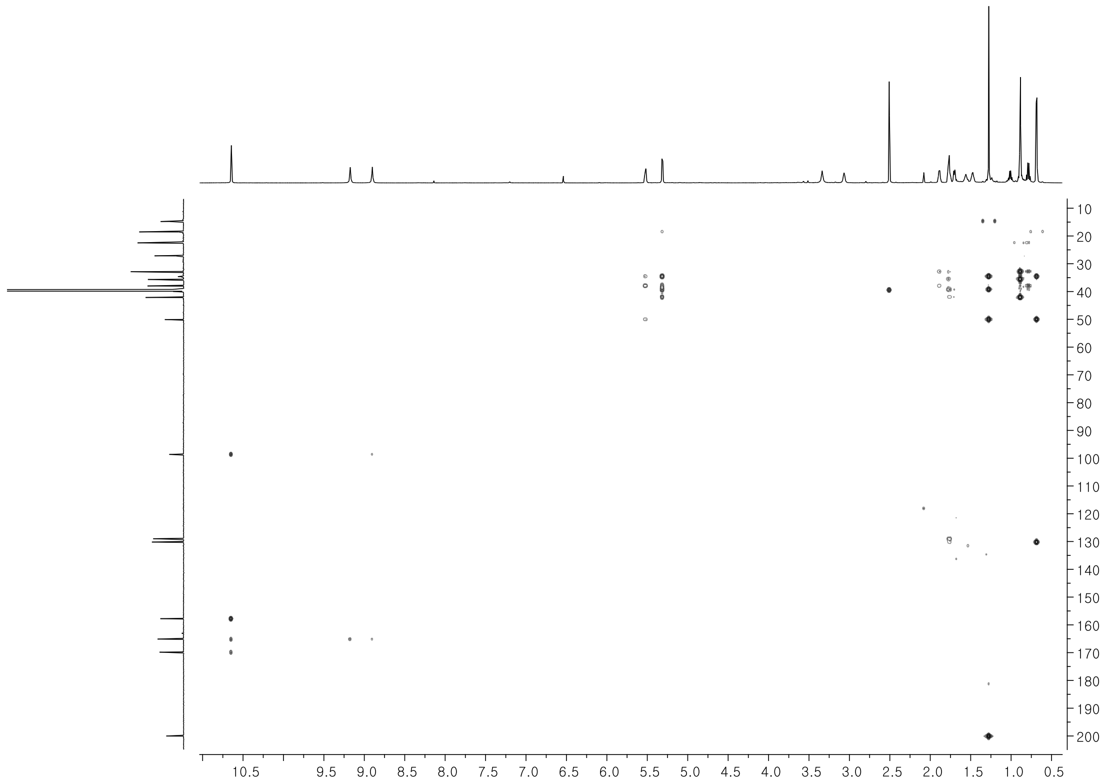

Figure S6. ROESY NMR spectrum (800 MHz) of ligiamycin A (**1**) in DMSO-d_6.

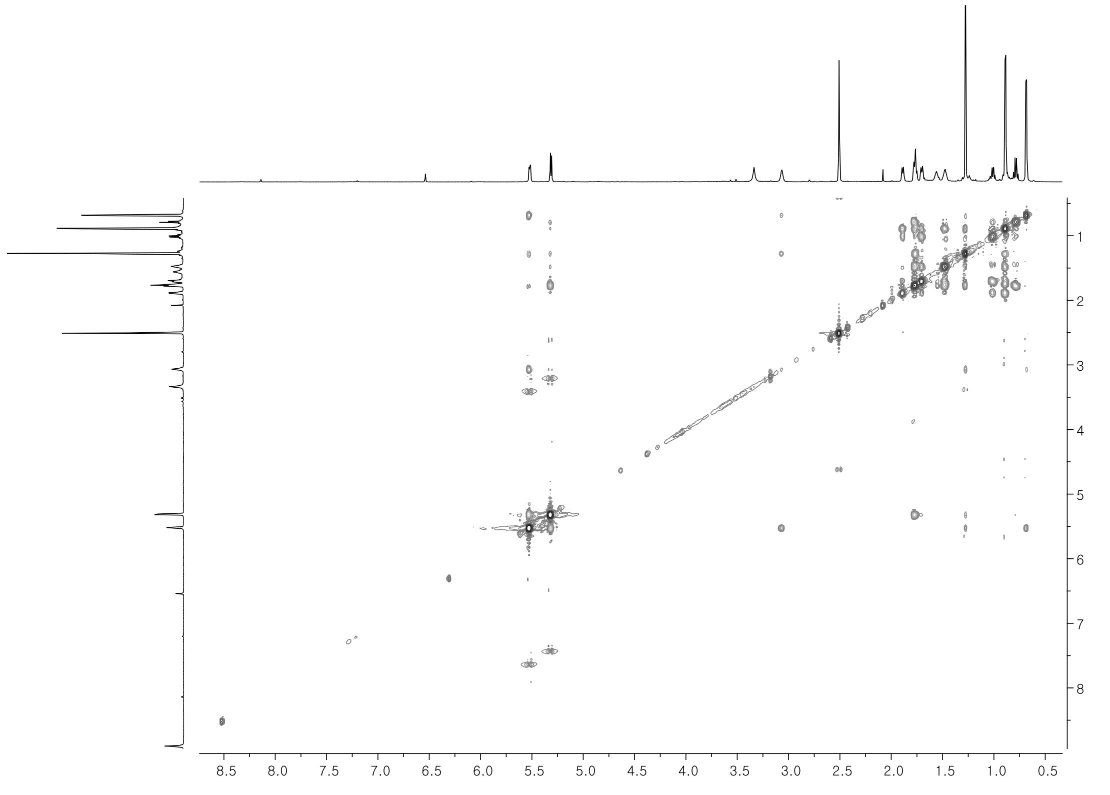

Figure S7. Expanded ROESY NMR spectrum (800 MHz) of ligiamycin A (**1**) in DMSO-d_6.

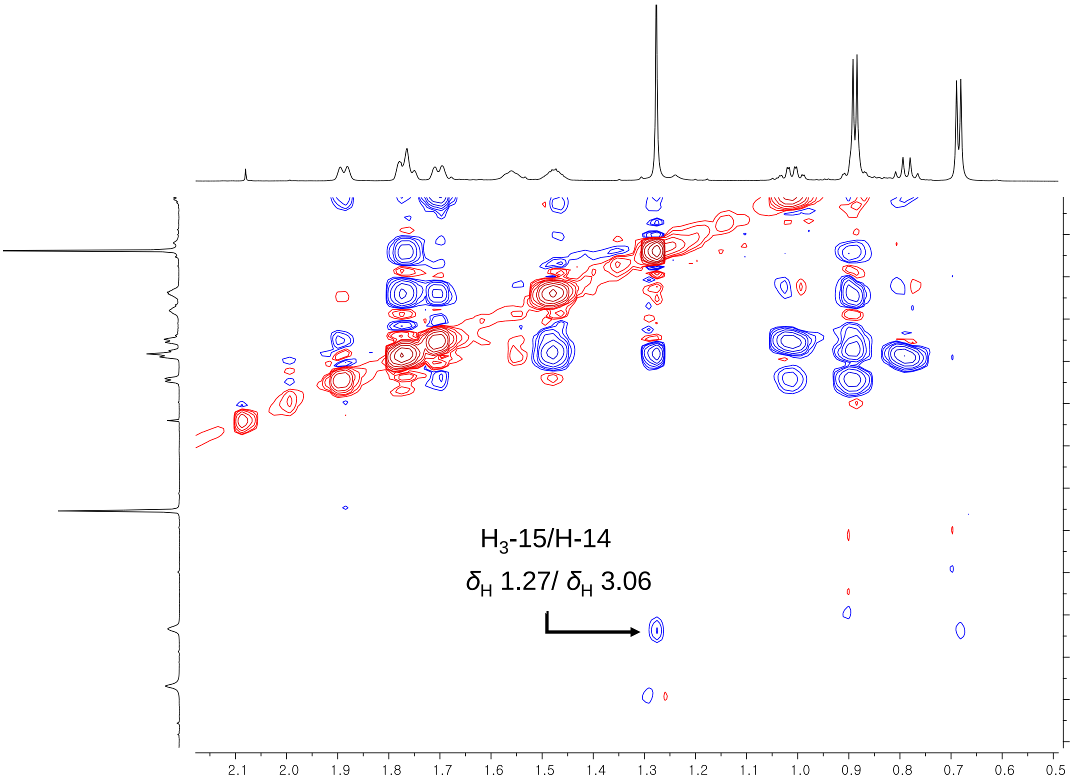

Figure S8. ^{1}H-^{15}N HSQC NMR spectrum (850 MHz) of ligiamycin A (**1**) in DMSO-d_6.

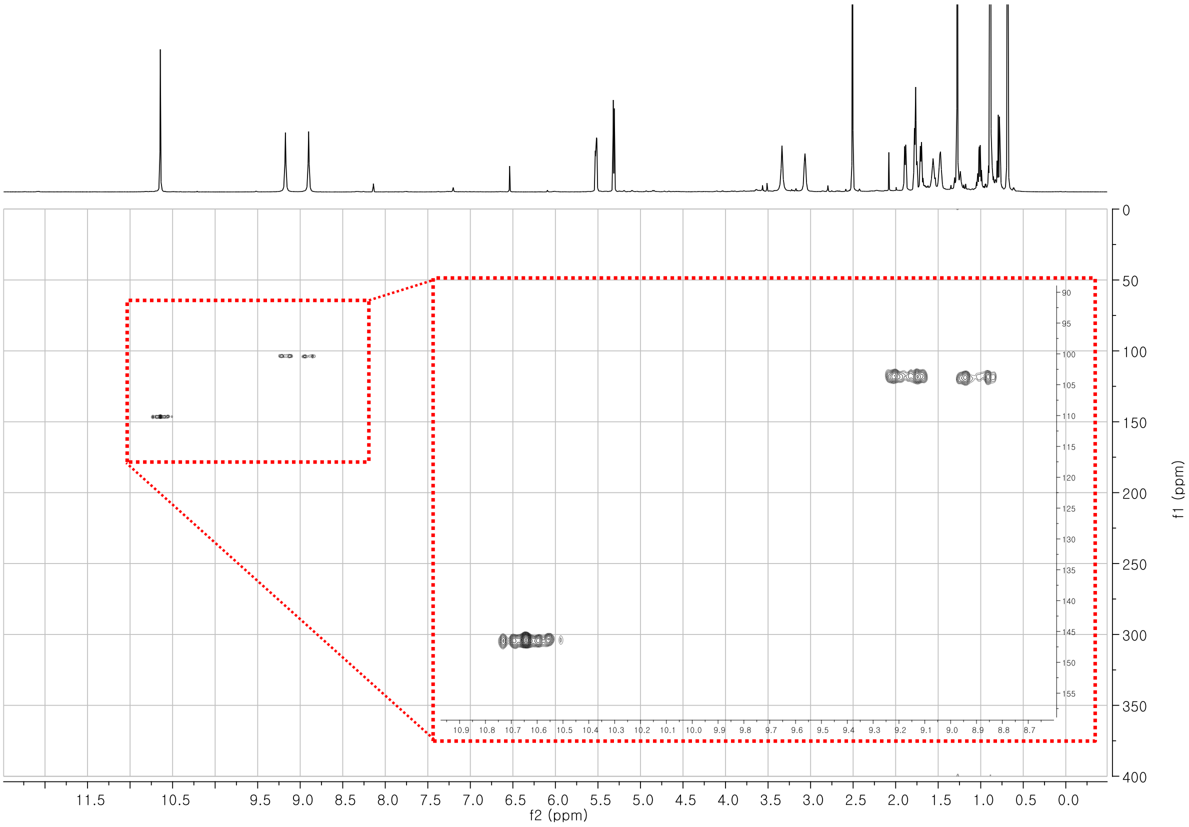

Figure S9. ^{1}H NMR spectrum (800 MHz) of ligiamycin B (**2**) in DMSO-d_6.

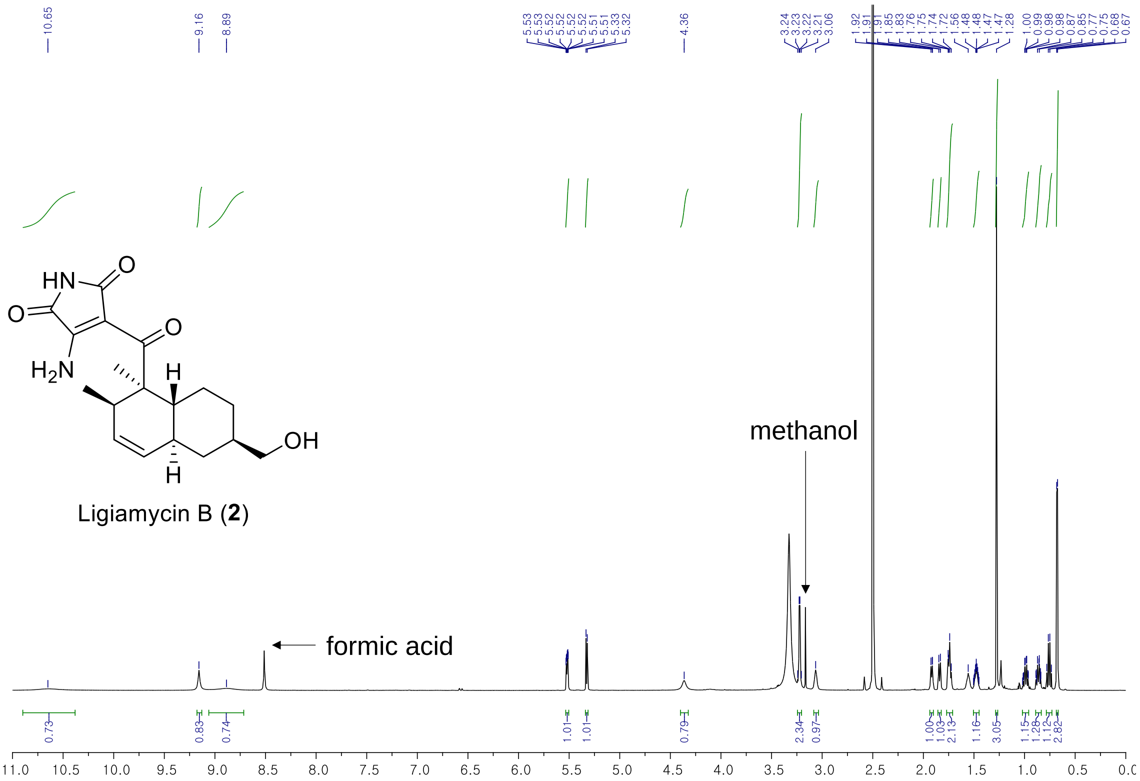

Figure S10. ^{13}C NMR spectrum (200 MHz) of ligiamycin B (**2**) in DMSO-d_6.

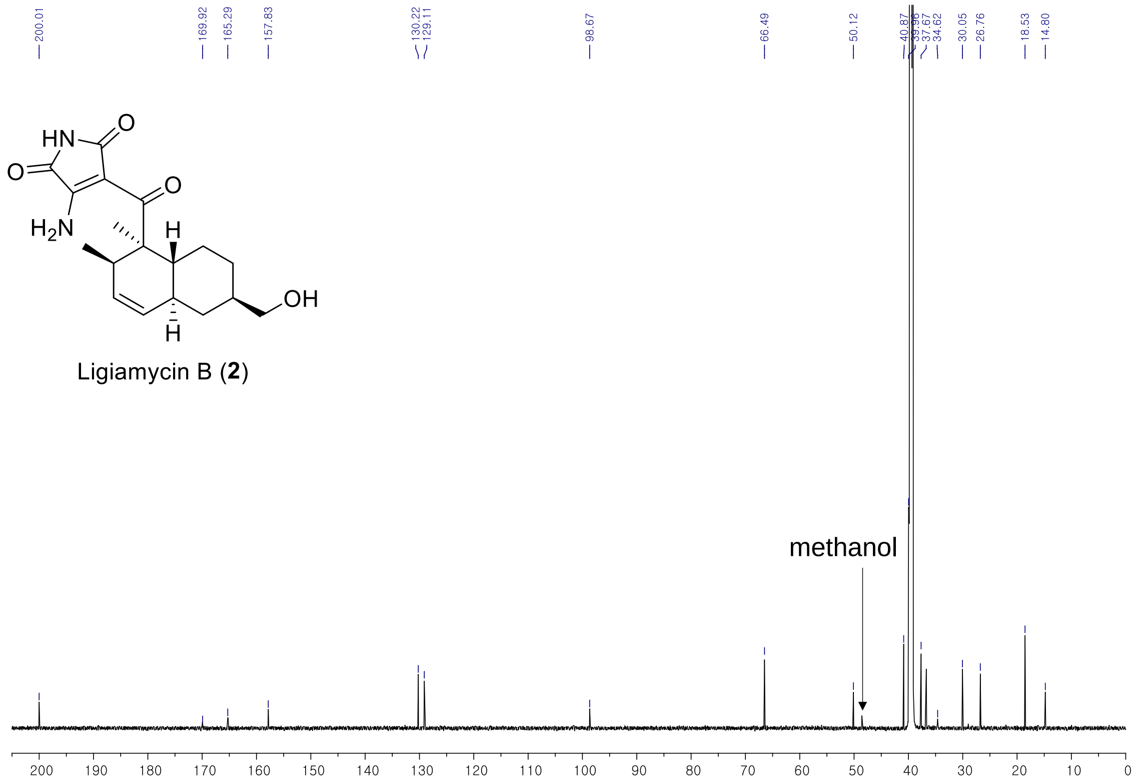

Figure S11. COSY NMR spectrum (800 MHz) of ligiamycin B (**2**) in DMSO-d_6.

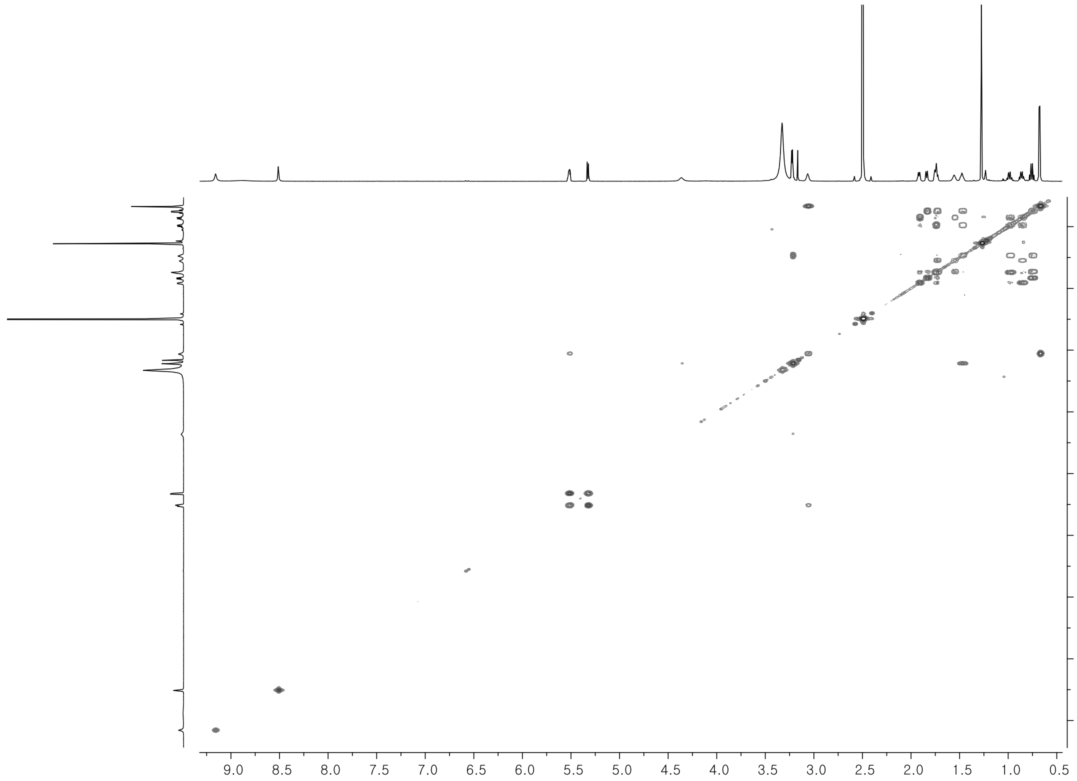

Figure S12. HSQC NMR spectrum (800 MHz) of ligiamycin B (**2**) in DMSO-d_6.

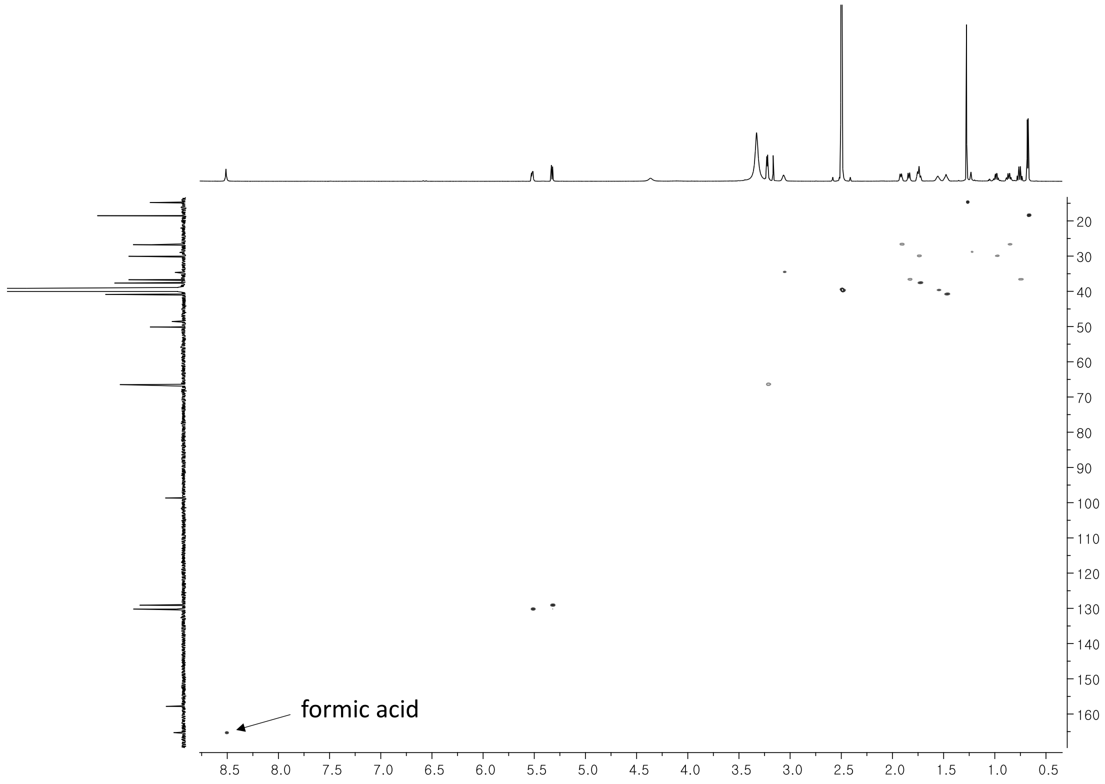

Figure S13. HMBC NMR spectrum (800 MHz) of ligiamycin B (**2**) in DMSO-*d*6.

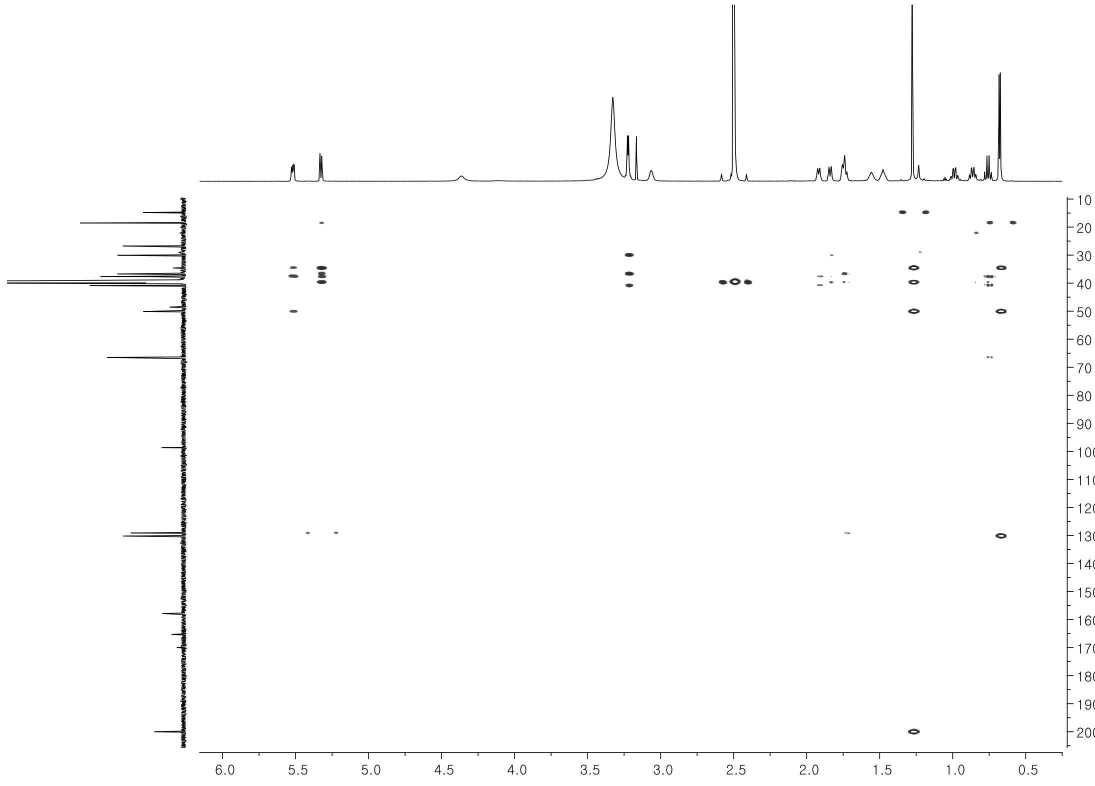

Figure S14. ROESY NMR spectrum (800 MHz) of ligiamycin B (**2**) in DMSO-*d*6.

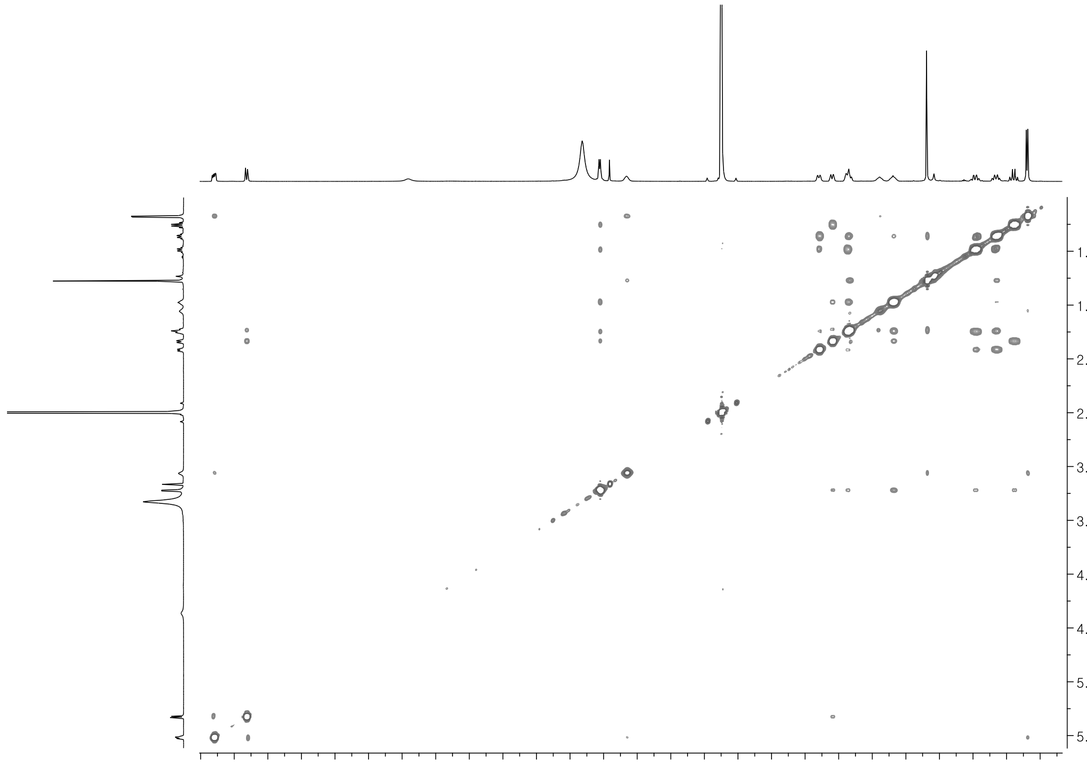

Figure S15. HR-ESI-MS data of ligiamycin A (**1**).

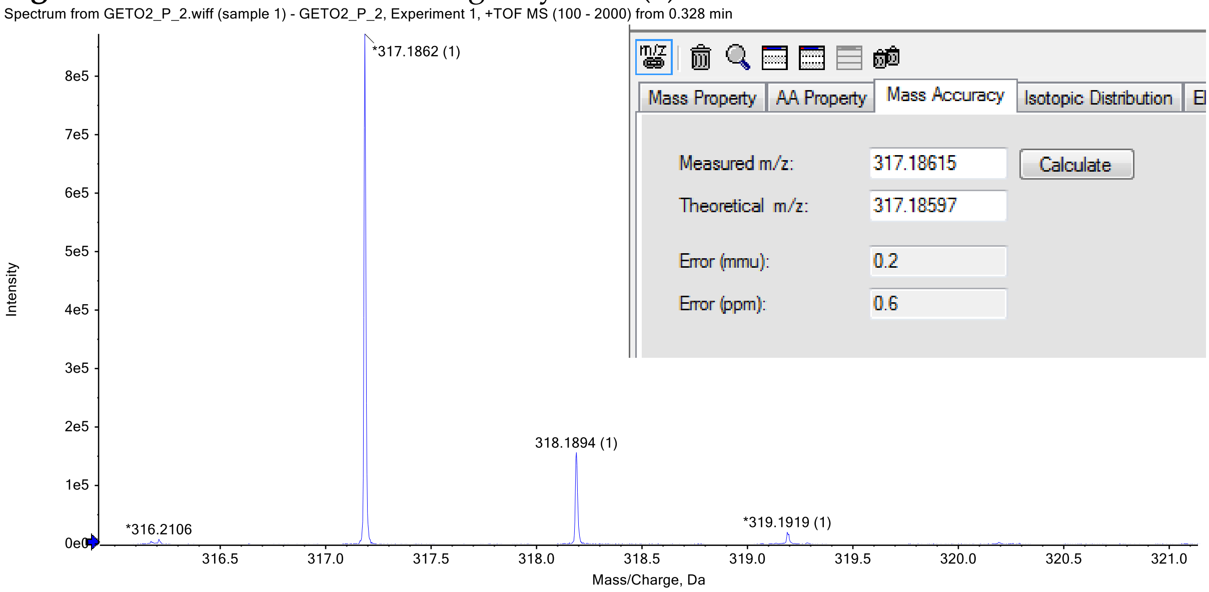

Figure S16. HR-FAB-MS data of ligiamycin B (**2**).

Data : FAB502 Date : 12-Jan-2020 21:46
Instrument : MStation
Sample : GET02
Note : m-NBA
Inlet : Direct Ion Mode : FAB+
RT : 0.15 min Scan# : (6,9)
Elements : C 100/0, H 100/0, N 5/0, O 10/0
Mass Tolerance : 1000ppm, 5mmu if m/z < 5, 10mmu if m/z > 10
Unsaturation (U.S.) : -0.5 - 15.0

	Observed m/z	Int%	Err[ppm / mmu]	U.S.	Composition
1	333.1816	1.61	-7.5 / -2.5	12.0	C21 H23 N3 O
2			-11.6 / -3.9	11.5	C23 H25 O2
3			+26.2 / +8.7	12.0	C22 H23 N O2
4			+4.5 / +1.5	8.0	C16 H23 N5 O3
5			+0.5 / +0.2	7.5	C18 H25 N2 O4
6			-25.2 / -8.4	3.0	C14 H27 N3 O6
7			+12.6 / +4.2	3.5	C13 H25 N4 O6
8			-29.2 / -9.7	2.5	C16 H29 O7
9			+8.5 / +2.8	3.0	C15 H27 N O7

Table S1. Composition of isolation agar media

	Composition of 1 L of distilled water
K	soluble starch 25 g, soytone 15 g, yeast extract 2 g, calcium carbonate 4 g
Actinomycete isolation agar	actinomycete isolation agar 22 g, glycerol 5 mL
modified K	yeast extract 3 g, glucose 2 g, mannitol 2 g, malt extract 5 g, soluble starch 5 g, soytone 5 g, calcium carbonate 1 g, agar 18 g
M4	agar 18 g
M2	glycerol 6 mL, arginine 1g, potassium dihydrogen phosphate 1 g, magnesium sulfate 0.5 g, agar 16 g
YPM	yeast extract 2 g, mannitol 4 g, peptone 2 g, agar 18 g
A1	soluble starch 10 g, yeast extract 2g, peptone 2 g, agar 18 g

- All isolation agar media contain cycloheximide 100 mg/L and sea salt 23 g/L.

Figure S17. Energy-minimized conformations of (a) ligiamycin A (**1**) and (b) ligiamycin B (**2**).

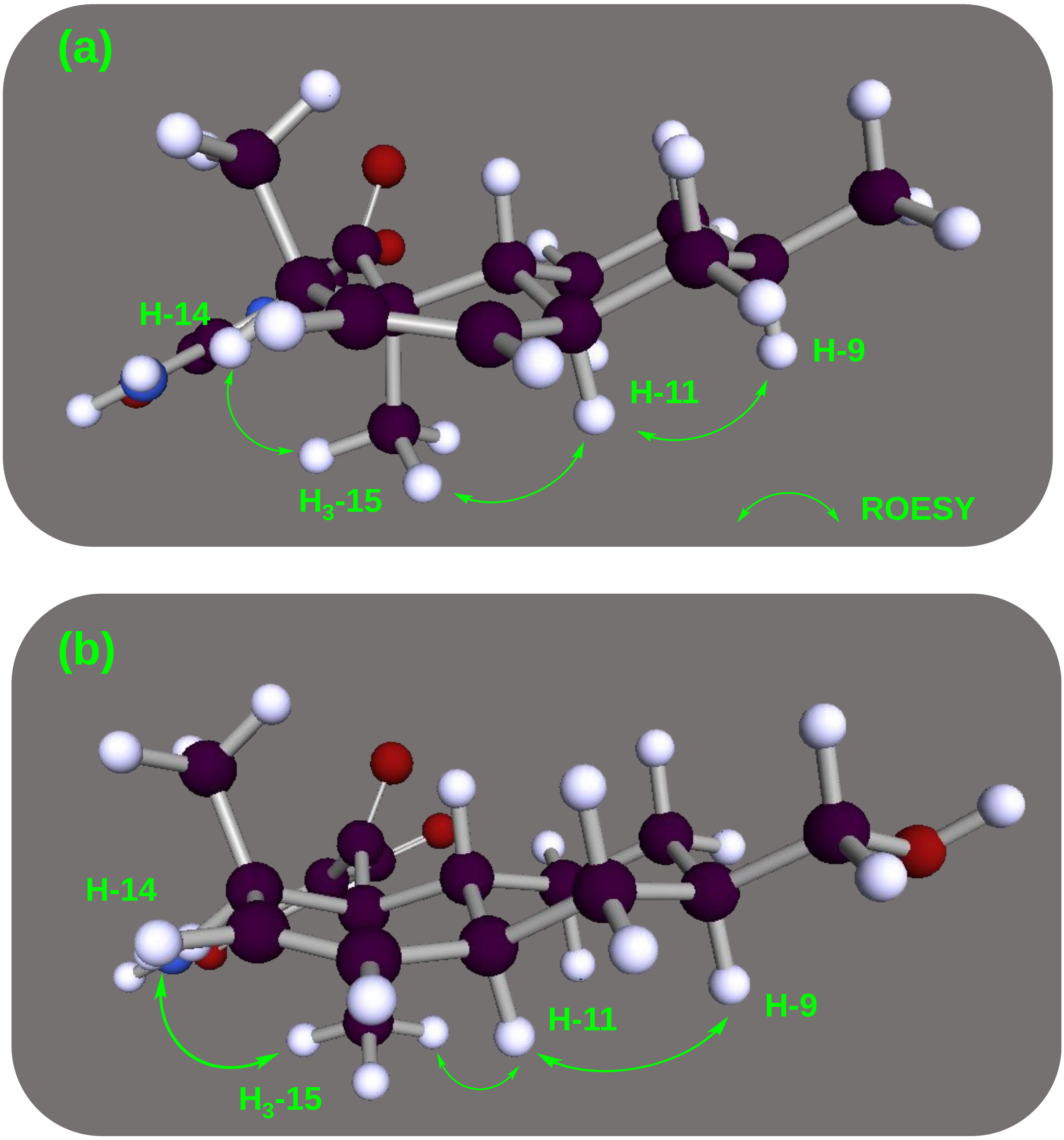

Figure S18. LC/MS traces (ion counts) of ion extraction for the ion $[M-H]^-$ at *m/z* 331 (ligiamycin B) (a) in a pure culture of *Streptomyces* sp. GET02.ST, (b) in a pure culture of *Achromobacter* sp. GET02.AC, and (c) in a co-culture. The LC/MS analyses were acquired with 10%–100% aqueous CH_3CN over 20 min.

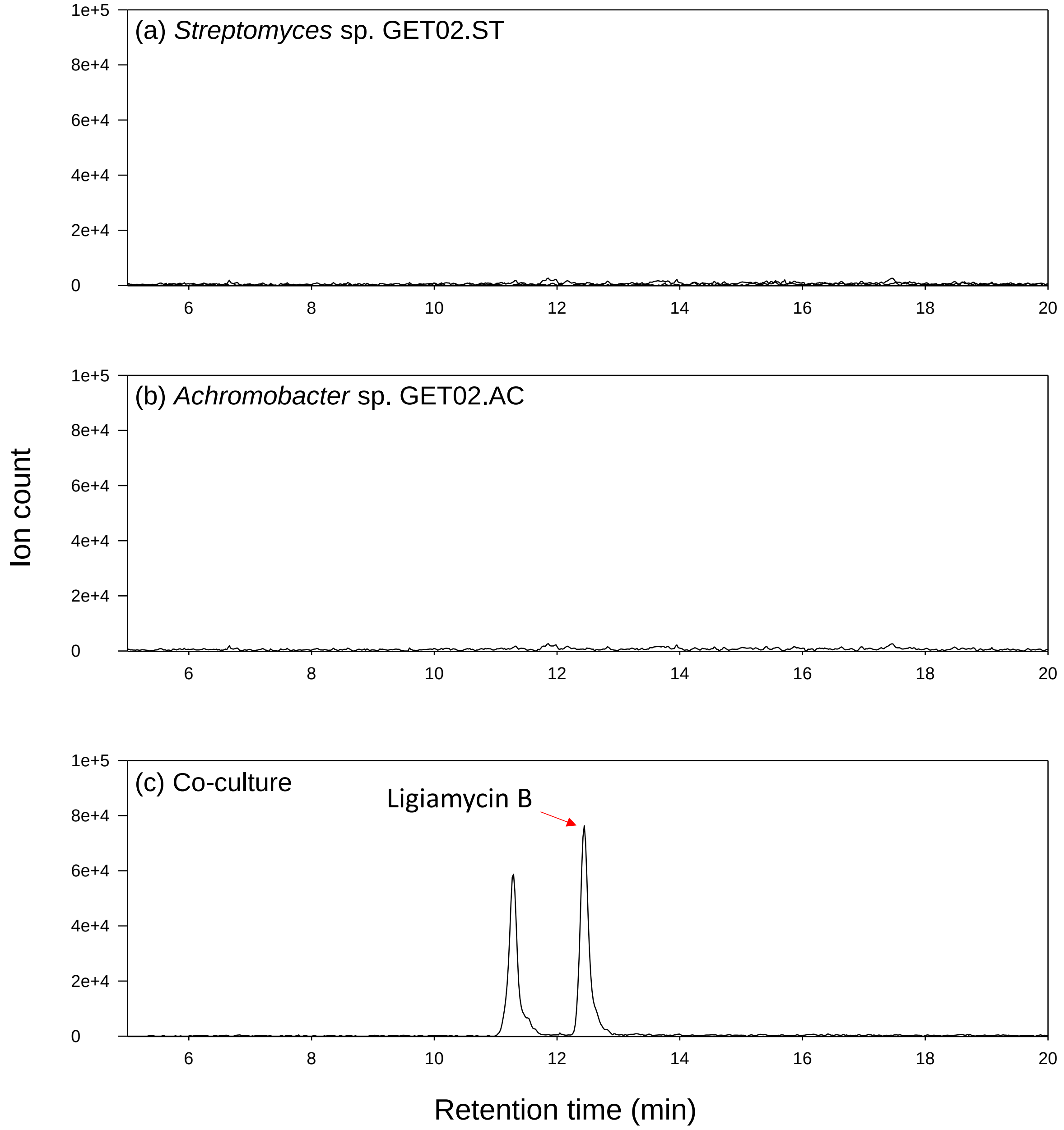

Table S2. Cartesian coordinates of ligiamycins A (**1**) and B (**2**).
(a) Cartesian coordinates of ligiamycin A (**1**).

No.	atom		Cartesian coordinates	
1	c	10.747191	1.99314333	5.74239902
2	c	12.7030904	0.52106201	4.02962159
3	c	11.5527494	-1.8272648	2.86988688
4	c	9.07494476	-2.3089731	2.72552631
5	c	7.08213678	-0.5719276	3.76559416
6	c	8.13517566	2.08515419	4.35128977
7	h	12.9047248	-3.1799318	2.06294629
8	h	8.42951044	-4.0689011	1.83408814
9	c	4.75572025	-0.3636018	2.0215213
10	h	6.38793542	-1.4154177	5.55931328
11	c	2.6515444	1.273216	3.18698917
12	h	5.34563144	0.4557426	0.18627568
13	h	4.01645079	-2.2769784	1.60782494
14	c	6.06110569	3.64872429	5.67496596
15	h	8.50145546	3.01794177	2.52055507
16	c	0.37764667	1.53001013	1.41219967
17	h	0.91737487	2.49388929	-0.3638737
18	h	-0.4096598	-0.3380548	0.90398064
19	h	-1.151038	2.64656724	2.29682578
20	c	10.5260226	0.64267948	8.31680471
21	h	9.87971043	-1.323049	8.06507838
22	h	12.3544908	0.56041396	9.31078282
23	h	9.17738222	1.60825586	9.57944162
24	c	14.0102437	2.09955363	1.95088888
25	h	14.2267381	-0.1635401	5.30141532
26	h	12.6174657	3.01922786	0.70400351
27	h	15.2276097	3.59747769	2.7413728
28	h	15.2070003	0.86007313	0.77049203
29	c	3.70571075	3.86299055	3.99060868
30	h	2.00211858	0.29626315	4.92478277
31	h	5.52495454	2.73742045	7.4809683
32	h	6.75073627	5.55771734	6.13600324
33	h	4.19412793	4.9638157	2.27656381
34	h	2.22135649	4.95018926	4.98313256
35	c	11.6606426	4.76862079	6.06147826
36	c	13.1643628	5.58774984	8.30820437
37	o	11.090653	6.36132775	4.50890435
38	c	12.5241215	8.01456227	9.59782512
39	n	14.4230566	8.41163014	11.4630844
40	c	16.1661521	6.50353642	11.4800426
41	c	15.3348643	4.69406929	9.40135746
42	o	18.0044816	6.22267483	12.8136178
43	o	10.7576825	9.41547212	9.29269867
44	h	14.4034461	9.8897984	12.6834271
45	n	16.8509791	2.69006851	9.04211366
46	h	18.4199617	2.57071403	10.1472989
47	h	16.5628832	1.4056492	7.65274903

(b) Cartesian coordinates of *ent*-ligiamycin A (**1**).

No.	atom	Cartesian coordinates		
1	c	-10.74782077	1.992968616	5.741281992
2	c	-12.70423105	0.519910931	4.029048342
3	c	-11.55292464	-1.825334103	2.863841968
4	c	-9.075156001	-2.306912236	2.719687814
5	c	-7.082271121	-0.571751059	3.763619661
6	c	-8.134779583	2.085293385	4.350496694
7	h	-12.90456457	-3.176442592	2.054107926
8	h	-8.429599934	-4.064621239	1.82435326
9	c	-4.75415717	-0.363106212	2.021538417
10	h	-6.390632663	-1.417516232	5.556938631
11	c	-2.649426828	1.271559131	3.189569221
12	h	-5.341758027	0.45784891	0.186791657
13	h	-4.01551296	-2.27652404	1.608296033
14	c	-6.060482954	3.646888482	5.676779602
15	h	-8.500627412	3.01947312	2.520735751
16	c	-0.375579292	1.53007731	1.414284525
17	h	-0.91549907	2.497279432	-0.359507025
18	h	0.409977825	-0.337556912	0.903034527
19	h	1.153587352	2.643896125	2.300779899
20	c	-10.52668505	0.641703992	8.315604972
21	h	-9.887443349	-1.325689364	8.061526566
22	h	-12.3534861	0.565109466	9.312364908
23	h	-9.172517084	1.602426241	9.575360643
24	c	-14.01853023	2.098866773	1.954757462
25	h	-14.22385718	-0.168998132	5.303049465
26	h	-12.63016678	3.026829426	0.709824562
27	h	-15.24154581	3.589300441	2.749953505
28	h	-15.21089983	0.857351124	0.772604165
29	c	-3.702706986	3.861116359	3.995261751
30	h	-1.999674063	0.292589455	4.925683624
31	h	-5.526837411	2.7345429	7.482538264
32	h	-6.749233288	5.556207086	6.136907106
33	h	-4.187588542	4.964611664	2.282459261
34	h	-2.219064262	4.944788224	4.992224877
35	c	-11.66168074	4.768923638	6.058981366
36	c	-13.16513515	5.590390156	8.305065129
37	o	-11.0909392	6.360506088	4.505228561
38	c	-12.52896332	8.021230912	9.589096287
39	n	-14.42570976	8.417060813	11.45677919
40	c	-16.16433152	6.505097798	11.4797315
41	c	-15.33179623	4.693706105	9.403530549
42	o	-18.00012807	6.222549111	12.81641419
43	o	-10.76648244	9.426194785	9.278539594
44	h	-14.40785942	9.897888417	12.67390188
45	n	-16.84272672	2.684961764	9.050505295
46	h	-18.41034673	2.564578054	10.15761278
47	h	-16.55504027	1.400110208	7.661317825

(c) Cartesian coordinates of ligiamycin B (**2**).

No.	atom	Cartesian coordinates		
1	c	10.75807943	1.983009671	5.725254648
2	c	12.7331079	0.502140684	4.042143753
3	c	11.59641083	-1.852929393	2.883186898
4	c	9.120490853	-2.334761778	2.712044759
5	c	7.116294463	-0.592417257	3.720845881
6	c	8.160640554	2.068814771	4.306720256
7	h	12.95732103	-3.211188852	2.101121171
8	h	8.485150847	-4.099976101	1.823637485
9	c	4.808960474	-0.398627823	1.949476916
10	h	6.400623161	-1.426724808	5.509818571
11	c	2.703297161	1.240509476	3.100613378
12	h	5.420969939	0.412042824	0.117772926
13	h	4.081289941	-2.317653318	1.541481711
14	c	6.069868981	3.636208798	5.601078438
15	h	8.546115692	2.994501617	2.476216363
16	c	0.444475193	1.453372766	1.318572174
17	h	1.027545213	2.48469895	-0.418903562
18	h	-0.156112126	-0.471878486	0.724561999
19	o	-1.54092207	2.72723682	2.581593788
20	c	10.50839127	0.647571891	8.30478839
21	h	9.863820083	-1.31938893	8.057941969
22	h	12.32553536	0.570234249	9.319505202
23	h	9.146009151	1.620939167	9.546268416
24	c	14.06185313	2.068348539	1.967770764
25	h	14.24338648	-0.174602857	5.333792207
26	h	12.68198447	2.979679367	0.700576231
27	h	15.26985149	3.571680481	2.761995842
28	h	15.27178225	0.82219267	0.807935034
29	c	3.732415429	3.841370225	3.892400854
30	h	2.009562598	0.273479833	4.824456065
31	h	5.516137995	2.734571159	7.406342739
32	h	6.752648144	5.548529211	6.059131852
33	h	4.242609375	4.923645446	2.171905468
34	h	2.224227272	4.918068774	4.846353488
35	c	11.66905394	4.760257404	6.037179698
36	c	13.14808746	5.593630787	8.295018996
37	o	11.11952022	6.342362924	4.466389748
38	c	12.49967724	8.03323621	9.556461661
39	n	14.37564446	8.439261615	11.44288181
40	c	16.11258772	6.526747078	11.49672368
41	c	15.3025741	4.703312964	9.422030656
42	o	17.93316023	6.251141086	12.85564628
43	o	10.74285391	9.438199062	9.216530678
44	h	14.34349027	9.925741905	12.65272332
45	n	16.81888636	2.693715171	9.097325001
46	h	18.37179954	2.578062111	10.22520485
47	h	16.54314587	1.396458038	7.717629474
48	h	-2.906916847	3.020370521	1.394554553

(d) Cartesian coordinates of *ent*-ligiamycin B (**2**).

No.	atom	Cartesian coordinates		
1	c	10.75807943	1.983009671	5.725254648
2	c	12.7331079	0.502140684	4.042143753
3	c	11.59641083	-1.852929393	2.883186898
4	c	9.120490853	-2.334761778	2.712044759
5	c	7.116294463	-0.592417257	3.720845881
6	c	8.160640554	2.068814771	4.306720256
7	h	12.95732103	-3.211188852	2.101121171
8	h	8.485150847	-4.099976101	1.823637485
9	c	4.808960474	-0.398627823	1.949476916
10	h	6.400623161	-1.426724808	5.509818571
11	c	2.703297161	1.240509476	3.100613378
12	h	5.420969939	0.412042824	0.117772926
13	h	4.081289941	-2.317653318	1.541481711
14	c	6.069868981	3.636208798	5.601078438
15	h	8.546115692	2.994501617	2.476216363
16	c	0.444475193	1.453372766	1.318572174
17	h	1.027545213	2.48469895	-0.418903562
18	h	-0.156112126	-0.471878486	0.724561999
19	o	-1.54092207	2.72723682	2.581593788
20	c	10.50839127	0.647571891	8.30478839
21	h	9.863820083	-1.31938893	8.057941969
22	h	12.32553536	0.570234249	9.319505202
23	h	9.146009151	1.620939167	9.546268416
24	c	14.06185313	2.068348539	1.967770764
25	h	14.24338648	-0.174602857	5.333792207
26	h	12.68198447	2.979679367	0.700576231
27	h	15.26985149	3.571680481	2.761995842
28	h	15.27178225	0.82219267	0.807935034
29	c	3.732415429	3.841370225	3.892400854
30	h	2.009562598	0.273479833	4.824456065
31	h	5.516137995	2.734571159	7.406342739
32	h	6.752648144	5.548529211	6.059131852
33	h	4.242609375	4.923645446	2.171905468
34	h	2.224227272	4.918068774	4.846353488
35	c	11.66905394	4.760257404	6.037179698
36	c	13.14808746	5.593630787	8.295018996
37	o	11.11952022	6.342362924	4.466389748
38	c	12.49967724	8.03323621	9.556461661
39	n	14.37564446	8.439261615	11.44288181
40	c	16.11258772	6.526747078	11.49672368
41	c	15.3025741	4.703312964	9.422030656
42	o	17.93316023	6.251141086	12.85564628
43	o	10.74285391	9.438199062	9.216530678
44	h	14.34349027	9.925741905	12.65272332
45	n	16.81888636	2.693715171	9.097325001
46	h	18.37179954	2.578062111	10.22520485
47	h	16.54314587	1.396458038	7.717629474
48	h	2.906917327	3.020369165	1.394553599

Table S3. ECD calculations of ligiamycins A (**1**) and B (**2**).
(a) ECD calculations of ligiamycin A (**1**).
total energy = -1034.1216
kinetic energy = 1024.30205
potential energy = -2058.4236

Parameters of Level DFT
DFT settings (Functional B3LYP / Gridsize M3)
Geometry optimization options (Energy 10^{-6} Hartree, Gradient norm ｜dE / dxyz｜= 10^{-3} Hartree/Bohr)

Energy minimized coordinates of ent.-donghaesulfin A at the basis set def2-TVZPP for all atoms (Å).

atomic coordinates			atom	charge
10.74748	1.993091	5.742177	c	6
12.70324	0.520975	4.029264	c	6
11.55283	-1.82752	2.869964	c	6
9.07499	-2.30907	2.725659	c	6
7.082228	-0.57168	3.765261	c	6
8.1354	2.085305	4.351122	c	6
12.90474	-3.18041	2.063312	h	1
8.42947	-4.06918	1.834638	h	1
4.755873	-0.36309	2.021141	c	6
6.387742	-1.415	5.558948	h	1
2.651623	1.273173	3.187232	c	6
5.345732	0.456912	0.186167	h	1
4.016772	-2.2764	1.60685	h	1
6.061362	3.649084	5.674628	c	6
8.501782	3.018072	2.520388	h	1
0.377367	1.5298	1.412878	c	6
0.916603	2.494027	-0.36316	h	1
-0.40968	-0.33834	0.904539	h	1
-1.15137	2.645953	2.297927	h	1
10.5263	0.642531	8.316544	c	6
9.87976	-1.3231	8.064764	h	1
12.3548	0.56001	9.310407	h	1
9.177843	1.60819	9.579302	h	1
14.0101	2.09925	1.950197	c	6
14.22707	-0.16342	5.30094	h	1
12.61715	3.018726	0.703353	h	1
15.22754	3.597292	2.740328	h	1
15.20673	0.859638	0.769811	h	1
3.705657	3.863056	3.990653	c	6
2.002742	0.295992	4.925106	h	1
5.525498	2.73818	7.48092	h	1
6.751008	5.558195	6.135179	h	1
4.193633	4.963952	2.276517	h	1
2.221344	4.950078	4.983441	h	1
11.66112	4.768497	6.061266	c	6
13.16448	5.587743	8.308205	c	6
11.09158	6.361131	4.508416	o	8

12.524	8.014522	9.597739	c	6
14.42246	8.41155	11.46344	n	7
16.16582	6.503761	11.48034	c	6
15.33476	4.694109	9.401758	c	6
18.00434	6.223311	12.81374	o	8
10.75764	9.415529	9.292174	o	8
14.40237	9.889458	12.68406	h	1
16.85065	2.689943	9.043098	n	7
18.41941	2.57057	10.14854	h	1
16.56265	1.405372	7.653894	h	1

(b) ECD calculations of *ent*-ligiamycin A (**1**).
total energy = -1034.1249
kinetic energy = 1024.30943
potential energy = -2058.4343

Parameters of Level DFT
DFT settings (Functional B3LYP / Gridsize M3)
Geometry optimization options (Energy 10^{-6} Hartree, Gradient norm | dE / dxyz | = 10^{-3} Hartree/Bohr)

Energy minimized coordinates of ent.-donghaesulfin A at the basis set def2-TVZPP for all atoms (Å).

atomic coordinates			atom	charge
-10.74763518	1.99302684	5.7415065	c	6
-12.70417649	0.52004045	4.02935204	c	6
-11.55301122	-1.82518565	2.8639768	c	6
-9.07527064	-2.30684428	2.71964991	c	6
-7.08225753	-0.57179715	3.76351562	c	6
-8.13460787	2.08523948	4.35070619	c	6
-12.9047504	-3.17621885	2.05428112	h	1
-8.42983514	-4.06456191	1.82424357	h	1
-4.75426033	-0.36307672	2.02128353	c	6
-6.39049255	-1.41770916	5.5567172	h	1
-2.64936248	1.27135221	3.18934535	c	6
-5.3419547	0.4581187	0.18667398	h	1
-4.0157486	-2.27648646	1.6077679	h	1
-6.06016272	3.64663737	5.67698983	c	6
-8.50044989	3.01958681	2.52102939	h	1
-0.37560819	1.52992843	1.41394775	c	6
-0.91557572	2.4973836	-0.35969053	h	1
0.40978932	-0.33769628	0.90241817	h	1
1.1536922	2.64353447	2.30047901	h	1
-10.52648889	0.6417964	8.31584514	c	6
-9.88734272	-1.32563119	8.06177811	h	1
-12.35325879	0.56530294	9.31266252	h	1
-9.17223732	1.60247431	9.57554218	h	1
-14.01854856	2.09912398	1.95520292	c	6
-14.22374241	-0.16889368	5.3034123	h	1
-12.63022997	3.02739022	0.71044776	h	1
-15.24172946	3.58934208	2.75055348	h	1

-15.21076231	0.85765676	0.77284408	h	1
-3.70243445	3.86087792	3.99541188	c	6
-1.99954975	0.29215396	4.92530704	h	1
-5.52648651	2.73413869	7.4826613	h	1
-6.74880375	5.55595542	6.1372874	h	1
-4.187297	4.96462656	2.28276802	h	1
-2.21867317	4.94431836	4.99245014	h	1
-11.66136655	4.76903758	6.0591666	c	6
-13.1652415	5.59053359	8.3049521	c	6
-11.09017018	6.36065317	4.50560901	o	8
-12.52963707	8.02165788	9.58873849	c	6
-14.42648556	8.41724852	11.45637247	n	7
-16.16481193	6.50502011	11.47934752	c	6
-15.33187751	4.69362925	9.4033017	c	6
-18.00063122	6.22225967	12.81594943	o	8
-10.76750924	9.42702934	9.27801922	o	8
-14.40874006	9.8979727	12.67362027	h	1
-16.84249741	2.68466145	9.05032746	n	7
-18.4101177	2.56408793	10.15740904	h	1
-16.55448529	1.39968848	7.66132321	h	1

(c) ECD calculations of ligiamycin B (**2**).
total energy = -1109.2367
kinetic energy = 1099.05832
potential energy = -2208.2951

Parameters of Level DFT
DFT settings (Functional B3LYP / Gridsize M3)
Geometry optimization options (Energy 10^{-6} Hartree, Gradient norm | dE / dxyz | = 10^{-3} Hartree/Bohr)

Energy minimized coordinates of ent.-donghaesulfin A at the basis set def2-TVZPP for all atoms (Å).

atomic coordinates			atom	charge
10.7579982	1.98374977	5.72483457	c	6
12.73321401	0.50293749	4.04180113	c	6
11.59672391	-1.85202557	2.88243125	c	6
9.12084058	-2.33410517	2.71144632	c	6
7.11656679	-0.59206392	3.72061422	c	6
8.16047192	2.06932095	4.30639752	c	6
12.95770294	-3.20974221	2.09951624	h	1
8.4856165	-4.09942252	1.82315732	h	1
4.80866918	-0.39947562	1.94995369	c	6
6.40161291	-1.42653776	5.5098038	h	1
2.70303088	1.24004892	3.10048793	c	6
5.42012378	0.41027638	0.11764556	h	1
4.08114213	-2.31879967	1.54313637	h	1
6.06953466	3.63654444	5.60061358	c	6
8.54581997	2.99497977	2.47585735	h	1
0.44400046	1.45243091	1.3186773	c	6
1.02665795	2.4834544	-0.41911109	h	1

-0.15664479	-0.47297157	0.72520828	h	1
-1.54121997	2.72643529	2.58187368	o	8
10.5081278	0.64798505	8.30417521	c	6
9.86355101	-1.31894305	8.05700415	h	1
12.32517417	0.57047507	9.31906531	h	1
9.14563683	1.6212019	9.54567112	h	1
14.06263844	2.0692073	1.96786799	c	6
14.2432519	-0.17402456	5.33363676	h	1
12.68316349	2.9808029	0.70044173	h	1
15.27063329	3.57230858	2.76252495	h	1
15.27269039	0.82300743	0.80819903	h	1
3.7318836	3.84109164	3.89211691	c	6
2.00932232	0.27328583	4.82452116	h	1
5.51603363	2.73495179	7.40599162	h	1
6.75202435	5.54900532	6.05846712	h	1
4.24197981	4.92309974	2.17140789	h	1
2.22360854	4.91782482	4.84587528	h	1
11.66915256	4.76097593	6.03733317	c	6
13.14808517	5.59370614	8.29553788	c	6
11.11959598	6.34346777	4.46700668	o	8
12.50063698	8.03382756	9.556651	c	6
14.37733725	8.43971698	11.44248941	n	7
16.11358723	6.5265263	11.49658229	c	6
15.30225461	4.70268975	9.42273177	c	6
17.93455533	6.25077959	12.85493121	o	8
10.7442729	9.43929076	9.21682785	o	8
14.34623027	9.92679307	12.65165419	h	1
16.81707455	2.69175427	9.09921531	n	7
18.36995058	2.57569523	10.22713836	h	1
16.54022993	1.39373929	7.72043106	h	1
-2.90715847	3.01991793	1.39486104	h	1

(d) ECD calculations of *ent*-ligiamycin B (**2**).
total energy = -1109.2367
kinetic energy = 1099.05832
potential energy = -2208.2951

Parameters of Level DFT
DFT settings (Functional B3LYP / Gridsize M3)
Geometry optimization options (Energy 10^{-6} Hartree, Gradient norm ｜dE / dxyz｜= 10^{-3} Hartree/Bohr)

Energy minimized coordinates of ent.-donghaesulfin A at the basis set def2-TVZPP for all atoms (Å).

atomic coordinates			atom	charge
-10.7579982	1.98374971	5.72483525	c	6
-12.73321429	0.50293657	4.04180276	c	6
-11.59672409	-1.85202856	2.88243692	c	6
-9.12084057	-2.33410658	2.71144979	c	6
-7.11656671	-0.59206289	3.72061341	c	6
-8.16047243	2.06932158	4.30639741	c	6

-12.95770305	-3.20974758	2.09952588	h	1
-8.48561627	-4.09942505	1.82316308	h	1
-4.80867161	-0.39947232	1.94994994	c	6
-6.40160938	-1.42653553	5.50980213	h	1
-2.70303172	1.24004871	3.10048588	c	6
-5.42012795	0.41028385	0.11764428	h	1
-4.08114558	-2.31879574	1.54312779	h	1
-6.06953524	3.63654519	5.60061365	c	6
-8.54582116	2.99498065	2.47585755	h	1
-0.44400163	1.45243239	1.31867503	c	6
-1.02665808	2.48346213	-0.41910999	h	1
0.15664022	-0.47296927	0.72519996	h	1
1.54122133	2.72642973	2.58187452	o	8
-10.50812698	0.64798611	8.30417642	c	6
-9.86355092	-1.31894232	8.05700606	h	1
-12.32517316	0.57047725	9.31906713	h	1
-9.14563533	1.62120318	9.5456715	h	1
-14.06263509	2.06920425	1.96786562	c	6
-14.24325389	-0.17402252	5.333638	h	1
-12.68315794	2.98079646	0.70043933	h	1
-15.27062939	3.57230797	2.76251882	h	1
-15.27268695	0.82300361	0.80819737	h	1
-3.73188186	3.84109098	3.89211982	c	6
-2.00932324	0.27328175	4.82451695	h	1
-5.51603662	2.7349538	7.40599308	h	1
-6.7520245	5.54900672	6.058465	h	1
-4.24197423	4.92310342	2.17141239	h	1
-2.22360614	4.91781992	4.84588195	h	1
-11.66915305	4.76097565	6.0373333	c	6
-13.14808549	5.59370533	8.29553833	c	6
-11.11959659	6.34346753	4.46700699	o	8
-12.50063594	8.033826	9.5566522	c	6
-14.37733617	8.43971565	11.44249073	n	7
-16.11358793	6.52652638	11.4965821	c	6
-15.30225606	4.70269008	9.42273095	c	6
-17.934557	6.25078094	12.85492995	o	8
-10.74427144	9.43928851	9.21682911	o	8
-14.34622833	9.92679114	12.65165628	h	1
-16.8170776	2.69175594	9.09921278	n	7
-18.36995416	2.57569747	10.22713525	h	1
-16.5402333	1.39374142	7.72042793	h	1
2.90715925	3.01991506	1.39486189	h	1

Figure S19. Comparison of the experimental ECD data of ligiamycin A (**1**) with the calculated ECD data; black line: experimental ECD data; blue dotted line: calculated ECD data (5*S*, 6*S*, 9*S*, 11*R*, and 14*S*); red dotted line: calculated ECD data (5*R*, 6*R*, 9*R*, 11*S*, and 14*R*).

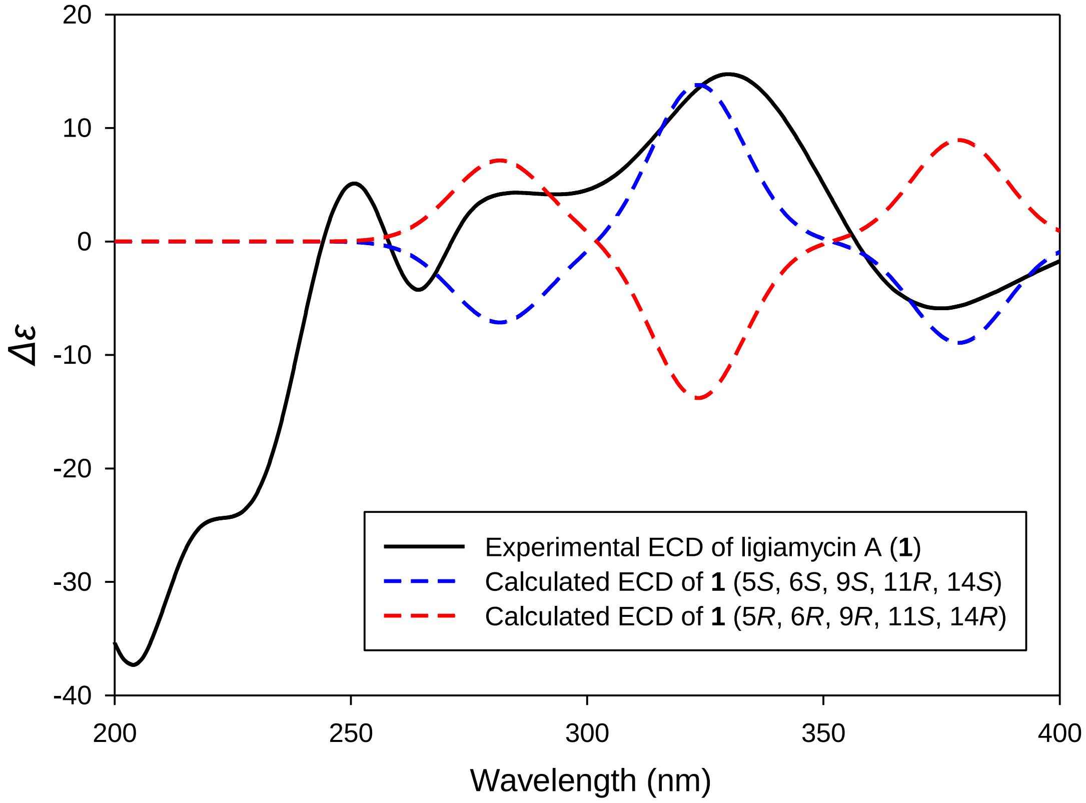

Figure S20. Comparison of the experimental ECD data of ligiamycin B (**2**) with the calculated ECD data; black line: experimental ECD data; blue dotted line: calculated ECD data (5*S*, 6*S*, 9*S*, 11*R*, 14*S*); red dotted line: calculated ECD data (5*R*, 6*R*, 9*R*, 11*S*, 14*R*).

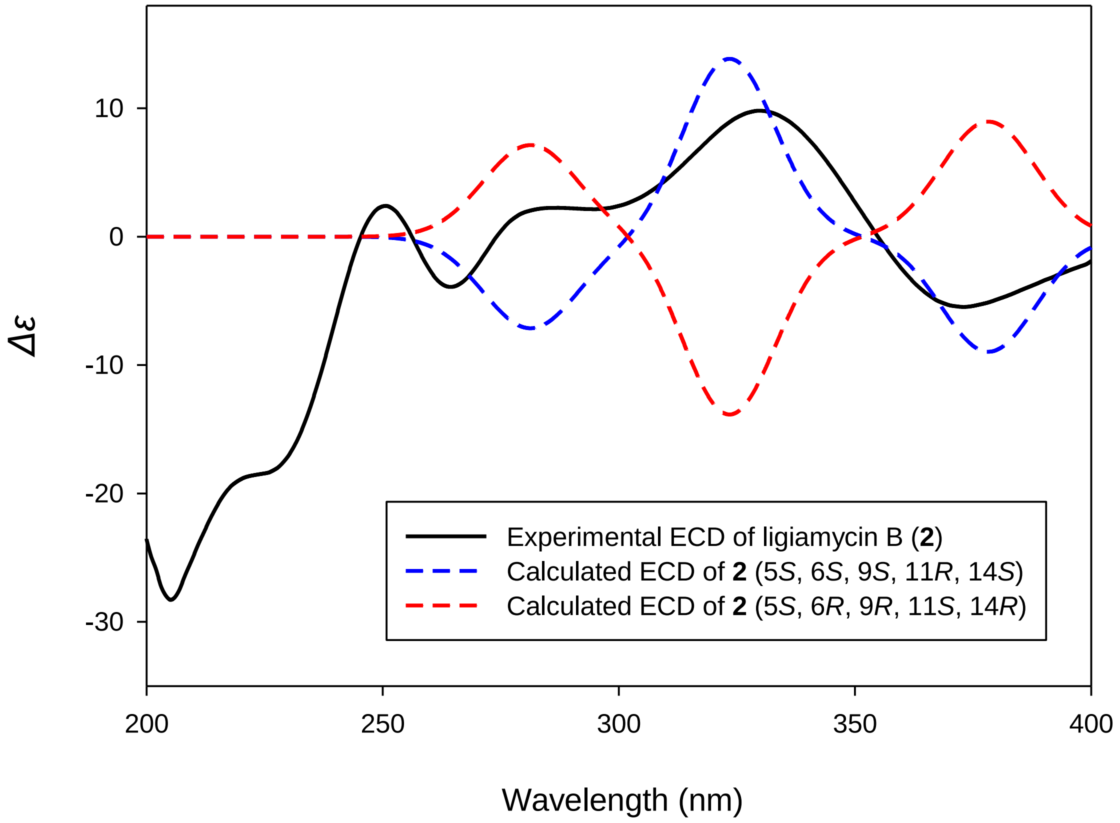

Proposed biosynthetic pathway of ligiamycins A (1) and B (2).

The core structures of ligiamycins A (**1**) and B (**2**) could be biosynthesized by a polyketide synthase-nonribosomal peptide synthetase (PKS-NRPS) hybrid pathway like the oxaleimides [1]. The decalin moiety could be cyclized through Diels-Alder cyclization. Cyclization and dehydration could be occurred to form a five membered maleimide ring. A further amination step is proposed to produce ligiamycin A (**1**) and subsequent oxidation could convert ligiamycin A (**1**) to ligiamycin B (**2**) (Scheme S1).

Scheme S1. Proposed biosynthetic pathway of ligiamycins. PKS-NRPS domain abbreviations: KS: ketosynthase; DH: dehydratse; MT: methyltransferase; KR: ketoreductases; ACP: acyl carrier protein; C: condensation; A: adenylation; T: thiolation; R: reductase; ER: enoylreductase.

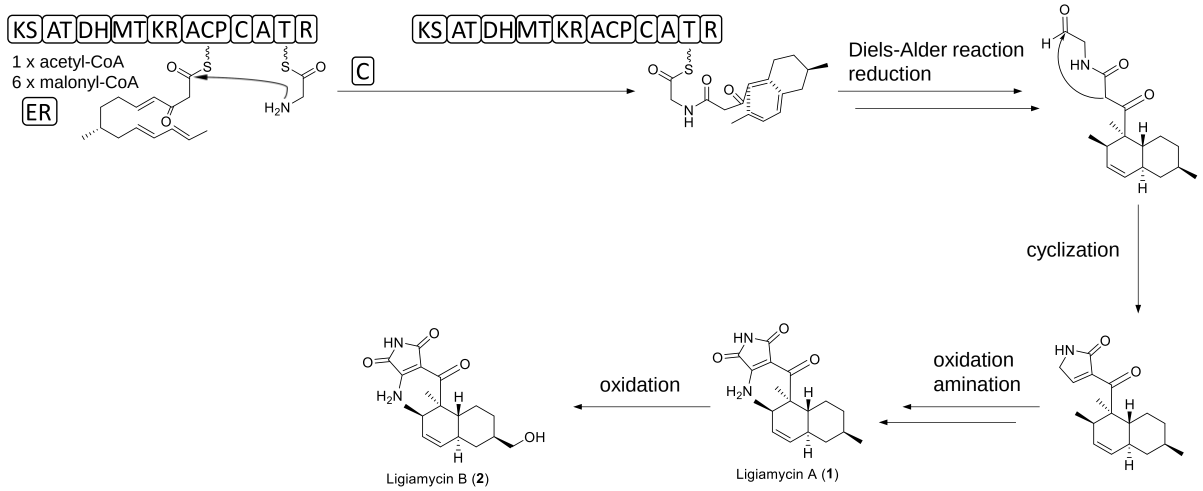

Reference

[1] Sato, M.; Dander, J.E.; Sato, C.; Hung, Y.-S.; Gao, S.-S.; Tang, M.-C.; Hang, L.; Winter, J.M.; Garg, N.K.; Watanabe, K.; et al. Collaborative biosynthesis of maleimide- and succinimide-containing natural products by fungal polyketide megasynthases. *J. Am. Chem. Soc.* **2017**, *139*, 5317–5320.
